# An Amidase_3 domain-containing *N-*acetylmuramyl-L-alanine amidase is required for mycobacterial cell division

**DOI:** 10.1038/s41598-017-01184-7

**Published:** 2017-04-25

**Authors:** Sibusiso Senzani, Dong Li, Ashima Bhaskar, Christopher Ealand, James Chang, Binayak Rimal, Chengyin Liu, Sung Joon Kim, Neeraj Dhar, Bavesh Kana

**Affiliations:** 1 DST/NRF Centre of Excellence for Biomedical TB Research, Faculty of Health Sciences, University of the Witwatersrand, National Health Laboratory Service, Johannesburg, 2001 South Africa; 2 Howard Hughes Medical Institute, Janelia Research Campus, Ashburn, Virginia USA; 3 Global Health Institute, Ecole Polytechnique Fédérale de Lausanne, Lausanne, Switzerland; 4 Baylor University, Department of Chemistry & Biochemistry, Waco, Texas USA; 5 Baylor University, Institute of Biomedical Studies, Waco, Texas USA; 6 Centre for the AIDS Programme of Research in South Africa, CAPRISA, Durban South Africa

## Abstract

Mycobacteria possess a multi-layered cell wall that requires extensive remodelling during cell division. We investigated the role of an amidase_3 domain-containing *N-*acetylmuramyl-L-alanine amidase, a peptidoglycan remodelling enzyme implicated in cell division. We demonstrated that deletion of MSMEG_6281 (Ami1) in *Mycobacterium smegmatis* resulted in the formation of cellular chains, illustrative of cells that were unable to complete division. Suprisingly, viability in the Δ*ami1* mutant was maintained through atypical lateral branching, the products of which proceeded to form viable daughter cells. We showed that these lateral buds resulted from mislocalization of DivIVA, a major determinant in facilitating polar elongation in mycobacterial cells. Failure of Δ*ami1* mutant cells to separate also led to dysregulation of FtsZ ring bundling. Loss of Ami1 resulted in defects in septal peptidoglycan turnover with release of excess cell wall material from the septum or newly born cell poles. We noted signficant accumulation of 3-3 crosslinked muropeptides in the Δ*ami1* mutant. We further demonstrated that deletion of *ami1* leads to increased cell wall permeability and enhanced susceptiblity to cell wall targeting antibiotics. Collectively, these data provide novel insight on cell division in actinobacteria and highlights a new class of potential drug targets for mycobacterial diseases.

## Introduction

During bacterial cell division, remodeling of the cell surface to create space for the insertion of new cell wall subunits, flagella, porins and specialized secretion apparatus is paramount for successful bacterial growth. This process is dynamic, involving the activity of a multitude of enzymes that act in a carefully coordinated manner to balance biogenesis versus degradation of cell wall polymers, such as peptidoglycan (PG). Dysregulation of these remodelling processes can result in cellular lysis or abnormal division that gives rise to non-viable progeny. As such, remodelling of the bacterial cell surface exposes numerous vulnerabilities that can be targeted for drug development. Mycobacteria represent a unique group of organisms within the actinomycetes that have a highly impermeable, complex cell wall with structurally distinct PG, arabinogalactan and mycolic acid layers^1, 2^.

During growth, mycobacterial cells extend through insertion of new cell wall material at the poles, followed by cell division in a manner contrasting to that of *Escherichia coli* and *Bacillus subtilis*, where cell extension occurs through insertion of new PG along the longitudinal axis of the cell via the activity of an enzyme complex termed the elongasome^3–7^. For separation of daughter cells, FtsZ, localizes at the division site, together with multiple cell wall synthetic and hydrolytic enzymes, which ultimately leads to constriction and cleavage of the septum^8^. In *E. coli*, expansion and the initiation of cell division are coordinated by enzymes such as MreB however, mycobacteria lack direct homologues of this central protein but retain enzymes such as DivIVA, a tropomyosin-like protein that plays a crucial role in cell elongation and determination of cell shape^9, 10^. Within the mycomembrane, remodelling of the PG during division involves a protein complex comprising resuscitation promoting factor B (RpfB), Rpf interacting protein A (RipA) and a high molecular weight penicillin binding protein (PonA1), which together regulate synthesis and degradation of septal PG^11–13^. In contrast, septal PG hydrolysis in other organisms is mediated by *N*-acetylmuramyl-L-alanine amidases, herein referred to as amidases, a group of zinc metalloenzymes responsible for hydrolysing the bond between the glycan strand and stem peptide in PG^14–16^. These enzymes are found in a wide range of organisms including bacteria, viruses and eukaryotes and can be further classified into either amidase_2 (PF01510) or amidase_3 (PF01520) – domain-containing enzymes^17^. *E. coli* has 5 amidases, which collectively play redundant roles in daughter cell separation, as evidenced by the formation of bacterial chains in the absence of two or more functional amidase genes, with associated defects in antibiotic resistance and PG recycling^14, 18–20^. Futher analysis identified two amidase activators, EnvC and NlpD, which directly interact with amidases to effect conformational changes, thus exposing the active site for PG hydrolysis^21, 22^. In *B. subtilis*, four cell wall amidases - CwlD, LytC, CwlC and CwlB - play a role in the formation of functional dormant spores and deletion of one of these resulted in either failure to form spores under stress conditions or the formation of spores that do not germinate^23–26^.

Despite their biological importance in other bacteria, the function of amidases in mycobacterial growth still requires further elucidation. The crystal structure of the mycobacterial amidase, Rv3717, retains overall similarity to other amidase_3 domain-containing amidases, and is able to cleave cross-linked PG/cell wall material from a variety of organisms^27, 28^. Biochemical analyses of Rv3915 (also designated CwlM) confirmed the presence of *N*-acetylmuramyl-L-alanine amidases activity^29^. Transposon mutagenesis identified this enzyme as essential for bacterial growth and depletion thereof results in reduced elongation of cells^30–32^. The biological activity of CwlM is mediated through phosphorylation by PknB and subsequent activation of MurA to coordinate PG synthesis at the cell pole during growth^31^. In *Mycobacterium tuberculosis*, Rv3717 has been implicated in bacillary dissemination to the spleen in the murine model of tuberculosis infection^33^. In this study, we characterise the function of an amidase_3 domain-containing amidase in *Mycobacterium smegmatis* and uncover an important role for this enzyme in mycobacterial growth.

## Results

### Amidase gene complement in *M. smegmatis* and *M. tuberculosis*

In prior work, we identified four putative PG degrading amidases in the genome of *Mycobacterium tuberculosis* and *M. smegmatis*, which were designated as Ami1 (Rv3717, MSMEG_6281), Ami2 (Rv3915- CwlM, MSMEG_6935), Ami3 (Rv3811, MSMEG_6406), and Ami4 (Rv3594, MSMEG_5315)^34^. Ami1 and Ami2 belong to the amidase_3 domain family, while Ami3 and Ami4 belong to the amidase_2 domain family of cell wall hydrolysing amidases. Amino acids essential for amidase_3 domain catalytic activity of AmiA in *Bacillus anthracis* 4229 include H341, E355, H415 and E486^35^. These residues are conserved in Ami1 however, in Ami2 both histidines have been replaced with arginine and the residue corresponding to E486 is replaced with an aspartate, Supplementary Fig. 1. Previous studies have confirmed biochemical activity in both Ami1 and Ami2^28, 29^ however, recent work indicates that amidase activity in Ami2 is relatively weak, suggesting that the amino acid variations in Ami2 affect catalytic activity^31^. For amidase_2 domains, structural analysis of AmiD from *E. coli* highlighted E104 and K159 as being essential for catalysis^36^, these residues are conserved in Ami4 but not in Ami3, where the glutamic acid is replaced by a proline and the lysine is replaced by threonine, Supplementary Fig. 1. Consequently, whilst Ami3 retains high similarity to amidase_2 domain containing enzymes, its catalytic activity requires confirmation. Further analysis of domain composition in the mycobacterial amidases revealed that Ami1 and Ami3 contain signal sequences to aid in translocation to the periplasm, Supplementary Fig. 2. In summary, there seems to be a differential distribution of signal peptides, catalytic residues and peptidoglycan binding domains between the four amidases in mycobacteria, conferring distinguishing features to each enzyme, suggestive of functional specialization. Considering the demonstrated biochemical activity of the amidase_3 domain containing enzymes in mycobacteria, we selected Ami1 for further analysis.

### Ami1 is required for cell separation during mycobacterial cell division

**Figure 1:**
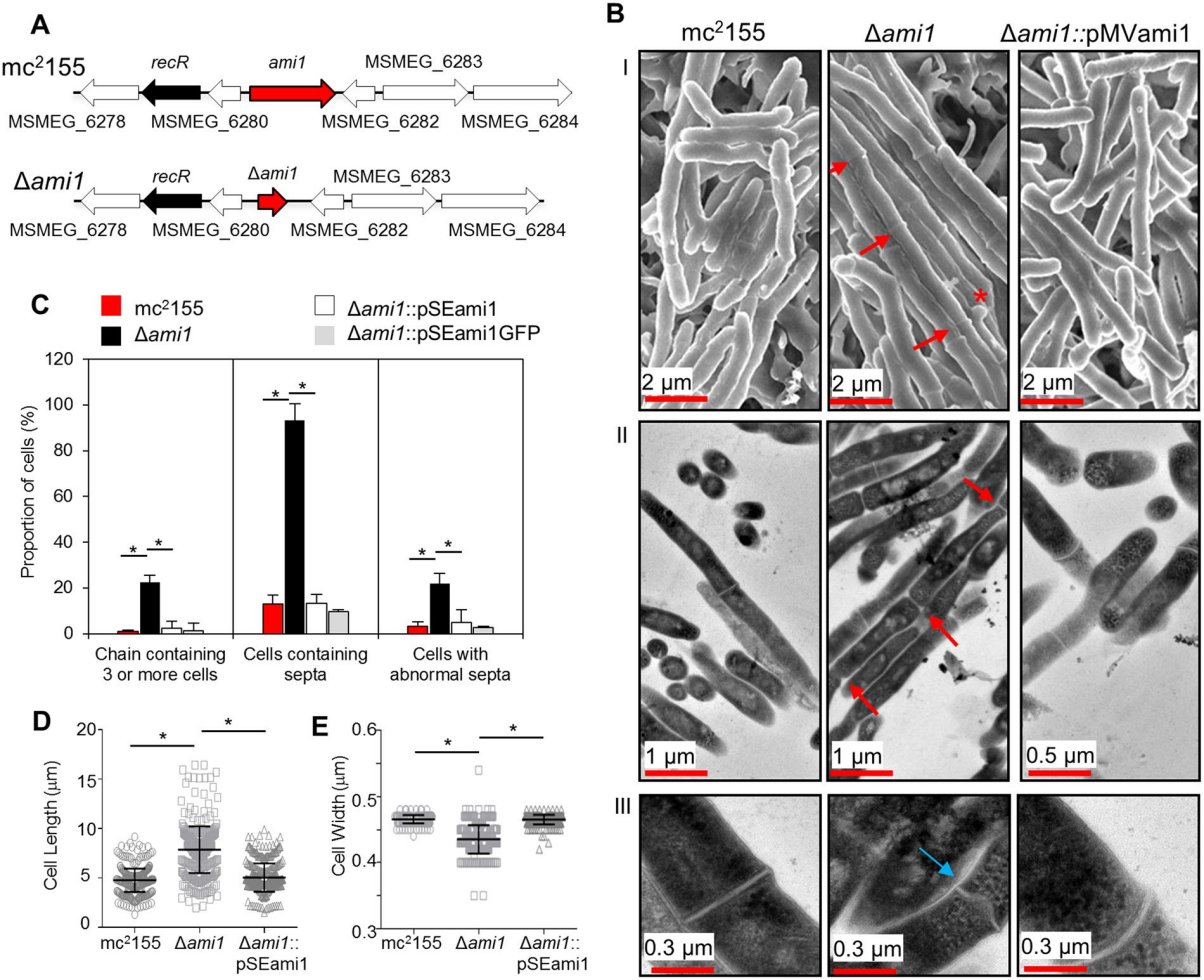
Phenotypic analysis of the Δ*ami1* mutant. (**A**) Genomic map of the Δ*ami1* deletion mutant. (**B**) (I) Scanning electron microscopy of the mc^2^155, Δ*ami1* and Δ*ami1*::pSEami1 strains depicting cell chaining, shown by the red arrows, as well as the occurrence of lateral buds (*). (II) and (III) Transmission electron microscopy to confirm synthesis of the septum in cell chains indicated by the red arrow as well as the formation of abnormal septa indicated by the blue arrow. (**C**) Histogram depicting the proportion of cells which chaining, arrested septation and aberrant septal structures (n = 400), *p < 0.0001. (**D,E**) scatter plots displaying the change in mean cell length and width respectively in the Δ*ami1* strain. Scale = 5 µm. Approximately 400 cells for each strain were assessed, p-values are the results of a student’s T-test.

To evaluate the physiological role of Ami1 in mycobacterial growth, the corresponding gene was deleted in *M. smegmatis* using two-step allelic exchange mutagenesis, Fig. 1A. The genotype of the strain was confirmed by PCR and Southern blot, Supplementary Fig. 3. Deletion of *ami1* did not affect growth kinetics in broth, sliding motility and colony morphology of *M. smegmatis*, confirming that it is dispensable for growth, Supplementary Fig. 4. However, analysis of cell morphology in the Δ*ami1* mutant by scanning and transmission electron microscopy revealed the formation of cellular chains consisting of numerous cells that failed to separate, Fig. 1B. Further analysis of ca. 400 cells indicated that 22% of the bacterial population examined displayed this phenotype, Fig. 1C. A notable increase in the frequency of cells possessing septa was also observed, as well as the presence of defective septa, indicative of arrested cell division in this strain, Fig. 1B,C. Due to the failure to separate, cellular chains consisting of 3 to 8 cells, with a cumulative size ranging from 2 to 16 µm in length, were observed in the Δ*ami1* mutant, Fig. 1D. Loss of *ami1* also led to a reduction in mean cell width, Fig. 1E, suggesting that defective cell separation in this case affects cell shape and width. These defects were reversed by genetic complementation confirming their association with loss of *ami1*. We next assessed localization of new PG synthesis in the Δ*ami1* strain using a BODIPY-vancomycin conjugate to spatially localize new PG subunits. Previous reports indicate that new PG synthesis is localized to the cell poles and/or septum^3^, a pattern, which is retained upon deletion of *ami1*, Supplementary Fig. 5. However, as cell separation is defective in the *ami1* mutant, we noted localization of new PG synthesis at the multiple septa present in this strain, Supplementary Fig. 5.

### Loss of Ami1 results in ectopic lateral bud and pole formation

**Figure 2:**
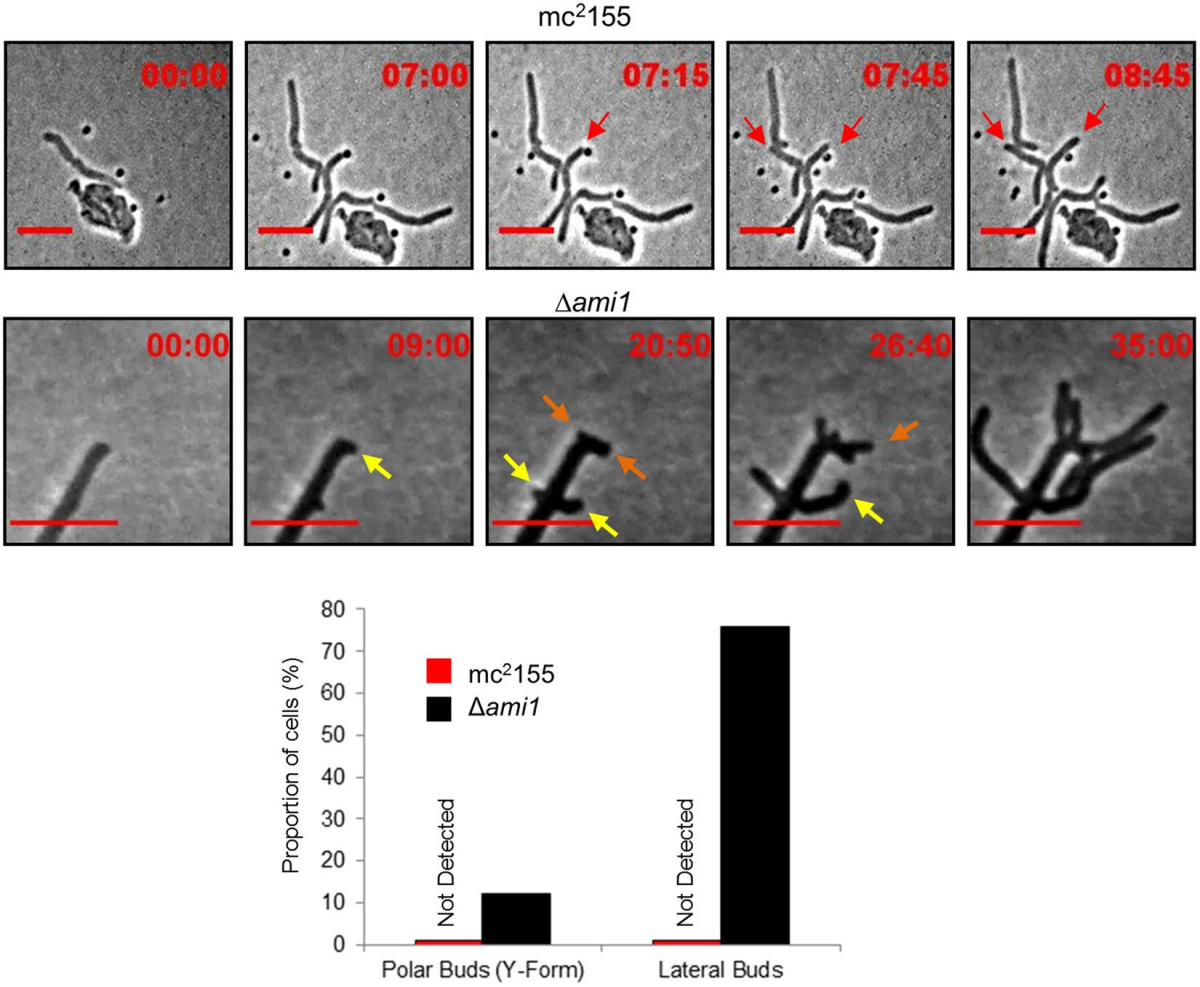
(**A**) Time-lapse microscopic analysis of growth in the Δ*ami1* mutant strain. Cells were grown and imaged in Middlebrook 7H9 media. Shown is the growth of mc^2^155 (top panel), which displays a growth and cell division pattern that is typical of mycobacteria with polar extension of cells (red arrows), as well as the Δ*ami1* strain, which exhibited the formation of lateral buds (yellow arrows) that subsequently formed branches. Scale = 5 µm. Ectopic polar growth and Y-formed cell division was also noted (orange arrows). Time frame is in hours. The histogram depicts the proportion of microcolonies with polar/lateral budding. For the Δ*ami1* mutant and wild type, 74 and 59 micro-colonies were assessed.

The striking cell division defects displayed by the Δ*ami1* mutant were incongruent with its apparent lack of any growth defects in broth culture, Supplementary Fig. 4. To further assess the effects of *ami1* deletion on cell division, we conducted time-lapse microscopy to monitor growth. Consistent with previous reports in wild type *M. smegmatis* ^4, 37^, cells elongate in a rod-shape, followed by division in all cells imaged, Fig. 2, Supplementary Movie 1 and Supplementary Fig. 6. In contrast, the Δ*ami1* mutant displayed formation of lateral buds at both the pole (observed for cells in 9 of 74 micro-colonies imaged) and the longitudinal axis (observed for cells in 56 of 74 micro-colonies imaged), which subsequently formed branches through cell elongation from the budding point, Fig. 2 and Supplementary Movie 2. These defects were only observed in the mutant and not in the wild type strain, Fig. 2. The extensive budding and ectopic polar growth shown in Supplementary Movie 2 was exceptionally extensive for this particular cell and further examples of these defects are given in Supplementary Fig. 6. We also noted Y-form cell division, which occurred either at the cell pole or lateral buds, Supplementary Fig. 6. Microscopic and single cell time-lapse analysis also identified a proportion of Δ*ami1* mutant cells that did not form lateral/polar buds and that were able to separate as evidenced by the lack of chaining. The occurrence of these cells suggests the presence of an alternate cell separation mechanism that is able to compensate for the loss of Ami1.

### Lateral budding and ectopic polar growth in the Δ*ami1* mutant is facilitated by mislocalization of the cell elongation machinery

**Figure 3:**
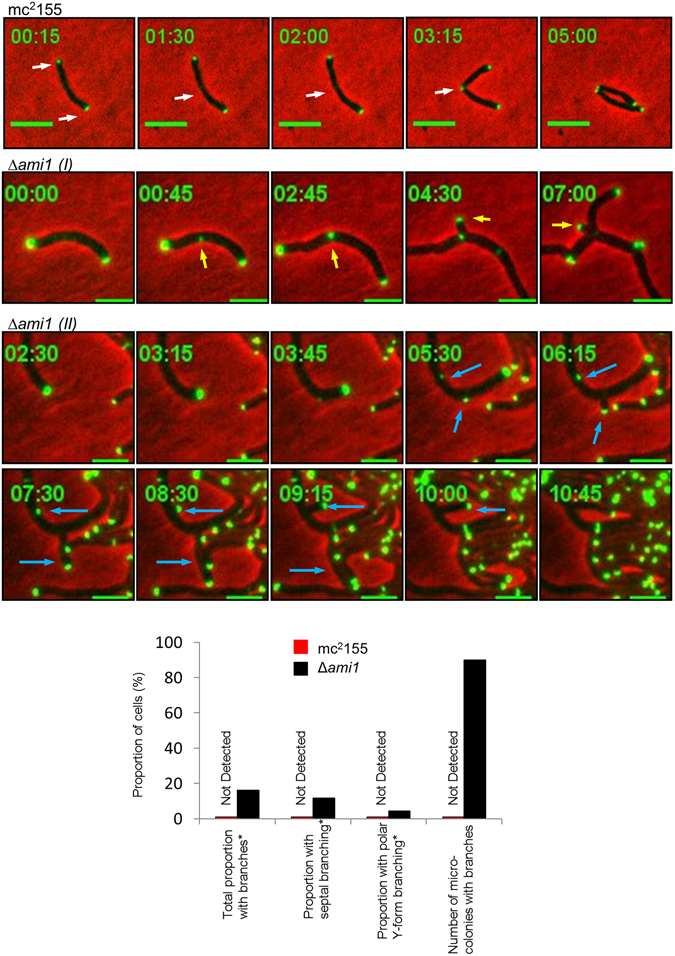
Time-lapse microscopic to assess the localization of DivIVA-rsEGFP. *M. smegmatis* mc^2^155 and Δ*ami1* cells were grown in Middlebrook 7H9 and subjected to time-lapse analysis to monitor DivIVA localization, indicated by white arrows in the wild type. In the Δ*ami* mutant (panel I), DivIVA localizes at midcell, but the failure to separate leads to the formation of ectopic cell poles, which extend into lateral buds (yellow arrow). Alternatively, in the Δ*ami* mutant (panel II), DivIVA traffics to the lateral axis of the cell and nucleates lateral branching (blue arrows). Scale bar = 5 µm. Time frame is in hours. The histogram depicts the proportion of cells with ectopic DivIVA localization. For the Δ*ami1* mutant and wild type, 1404 and 1626 individual cells were assessed. *The assessment of the proportion of cells with branches is subject to underestimation as cellular crowding makes accurate quantification difficult. Reported here is the proportion of cells with clearly visible branching patterns.

We noted that ectopic polar growth and lateral buds arose through the formation of functional cell poles, which proceeded to elongate in the case of lateral buds or ectopic cell poles. We reasoned that these aberrant growth modalities could arise through mislocalization of DivIVA, a key determinant in the nucleation and formation of functional cell poles in mycobacteria. Consistent with this hypothesis, we noted polar and septal localization of DivIVA in wild type cells as previously reported^38^, however, in the Δ*ami1* mutant the septal DivIVA foci flicker and become unstable, followed by formation of ectopic cell poles at the septum (Fig. 3, Supplementary Movie 3 [wild type] and Supplementary Movie 4 [Δ*ami1*]). This defect was observed in 165 out of 226 Δ*ami1* mutant cells that displayed branching. Surprisingly, we also noted DivIVA mislocalization to the lateral axis of the cell, which then allowed for ectopic pole formation along the cell axis (Fig. 3, panel II and Supplementary Movie 5). In 61 out of 226 Δ*ami1* mutant cells with branches, ectopic pole formation in the form of Y-form growth occurred in the polar region of the cell. A total of 45 out of 50 videos assessed for the Δ*ami1* mutant displayed one or more cells with ectopic DivIVA localization. These defects were not observed in the wild type strain.

### Bundling of FtsZ in the Δ*ami1* mutant

**Figure 4:**
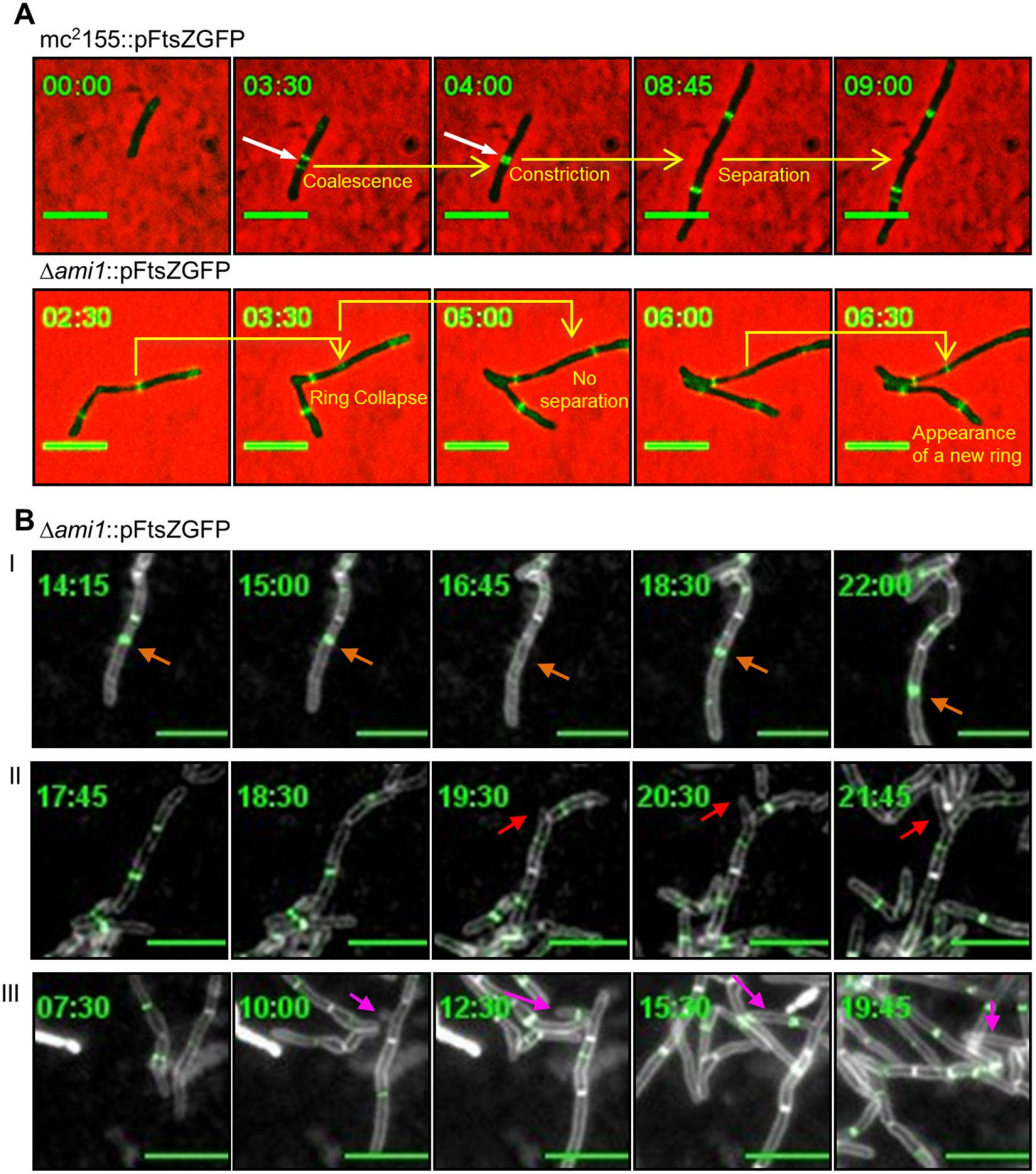
Time-lapse microscopic to assess the localization of FtsZ-rsEGFP. (**A**) *M. smegmatis* mc^2^155 and Δ*ami1* cells were grown in Middlebrook 7H9 and subjected to time-lapse analysis to monitor FtsZ bundling and constriction, indicated by white arrows. In the Δ*ami* mutant, FtsZ is able to form rings, but these eventually collapse. (**B**) Time-lapse microscopic to assess the localization of FtsZ-rsEGFP in cells stained with FM4-64. Cells were grown in Middlebrook 7H9 and stained with FM4-64 during time lapse analysis. White color indicates FM4-64 staining. Depicted in panel I is the formation of FtsZ rings in a single cellular compartment, followed by collapse and repolymerization. In panel II, FtsZ rings bundle in two connected daughter cells, but these do not coalese at the septum instead, failure to separate leads to the formation of a lateral bud at the septum (red arrows). In panel III, failure to separate leads to the formation of a lateral bud, not located at the septum, with subsequent formation of a FtsZ ring in the lateral bud (magenta arrows). Scale bar = 5 µm. Time frame is in hours.

The ability of the *ami1* defective mutant to continue cell division, albeit in an aberrant manner, required further investigation. The formation of cellular chains in the Δ*ami1* mutant suggested that cell elongation and synthesis of new septa continues in this strain however, the final steps of cell separation were defective. Hence, we next sought to analyze the cell division defects in the Δ*ami1* mutant by assessing the behavior of the essential cell division protein FtsZ, which localizes in rings at the septum and promotes invagination of the cell wall and subsequent cell separation. For this, we constructed a C-terminally tagged FtsZ-rsEGFP fusion protein. Using time-lapse microscopy, we found that FtsZ appeared as two distinct rings in wild type *M. smegmatis* and these eventually coalesced, followed by constriction and cell separation, Fig. 4A, Supplementary Movie 6 and further examples are given in Supplementary Fig. 7. In contrast, in the Δ*ami1* mutant, the appearance of double FtsZ rings was poorly coordinated or diffused and in many cases FtsZ was able to form rings but the appearance of rings was not always followed by cell separation, Fig. 4A. In the Δ*ami1* mutant, the two-ring coordination of FtsZ did not occur instead, often a single FtsZ ring was placed at the site of a future septum followed by failure to separate, Fig. 4A. In some cases, collapse of the FtsZ ring was observed. Further examples are given in the Supplementary Fig. 7 and Supplementary Movie 7. To further investigate the behavior of FtsZ-GFP in septal regions of the Δ*ami1* mutant, we studied FtsZ localization in cells stained with FM4-64, which allowed for a clearer focus on the septum. We noted that FtsZ rings form within individual cellular compartments and breakdown before moving to the septum and failure to separate leads to septal or lateral cell bud formation, Fig. 3B.

**Figure 5:**
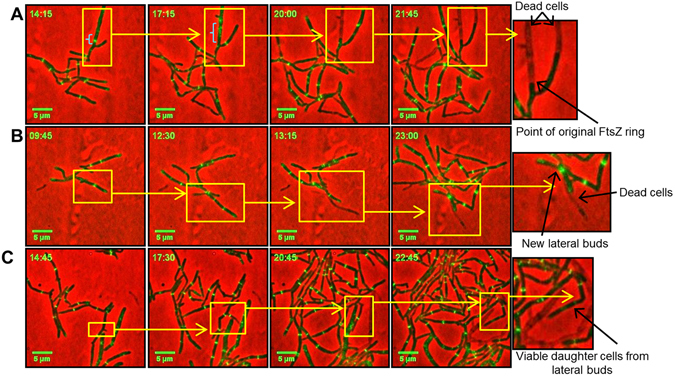
Time-lapse microscopic analysis of the Δ*ami1* mutant carrying FtsZ-rsEGFP. Cells were monitored for FtsZ bundling around lateral buds. (**A**) Video stacks depicting the progression of lateral buds (yellow rectangles), followed by death of the mother cell, at the point from which the original FtsZ ring was formed (blue parenthesis), and death of one daughter cell that resulted from the lateral bud. (**B**) Depicts the progression of two lateral buds, followed by death of one bud and half of the original mother cell. Thereafter, two new buds appeared over the husks of these dead cells. (**C**) Depicts the formation of a lateral bud that proceeded to form a viable daughter cell. Scale bar = 5 µm. Time frame is in hours.

Considering this aberrant behavior of FtsZ, we next sought to determine the fate of cells that fail to separate. Our analysis revealed that failure to separate often led to death of one daughter cell, Fig. 5. In addition, failure to separate after cytokinesis often led to lateral budding, which was followed by death of either one of the original cells (Fig. 5A) or the lateral bud (Fig. 5B), in the case of the latter, we also observed the formation of new lateral buds over the husks of dead cells. In some cases, the lateral bud continued to grow and divide with cell separation defects similar to that of the mother cell (Fig. 5C).

### Defective septal PG turnover in the Δ*ami1* mutant

**Figure 6:**
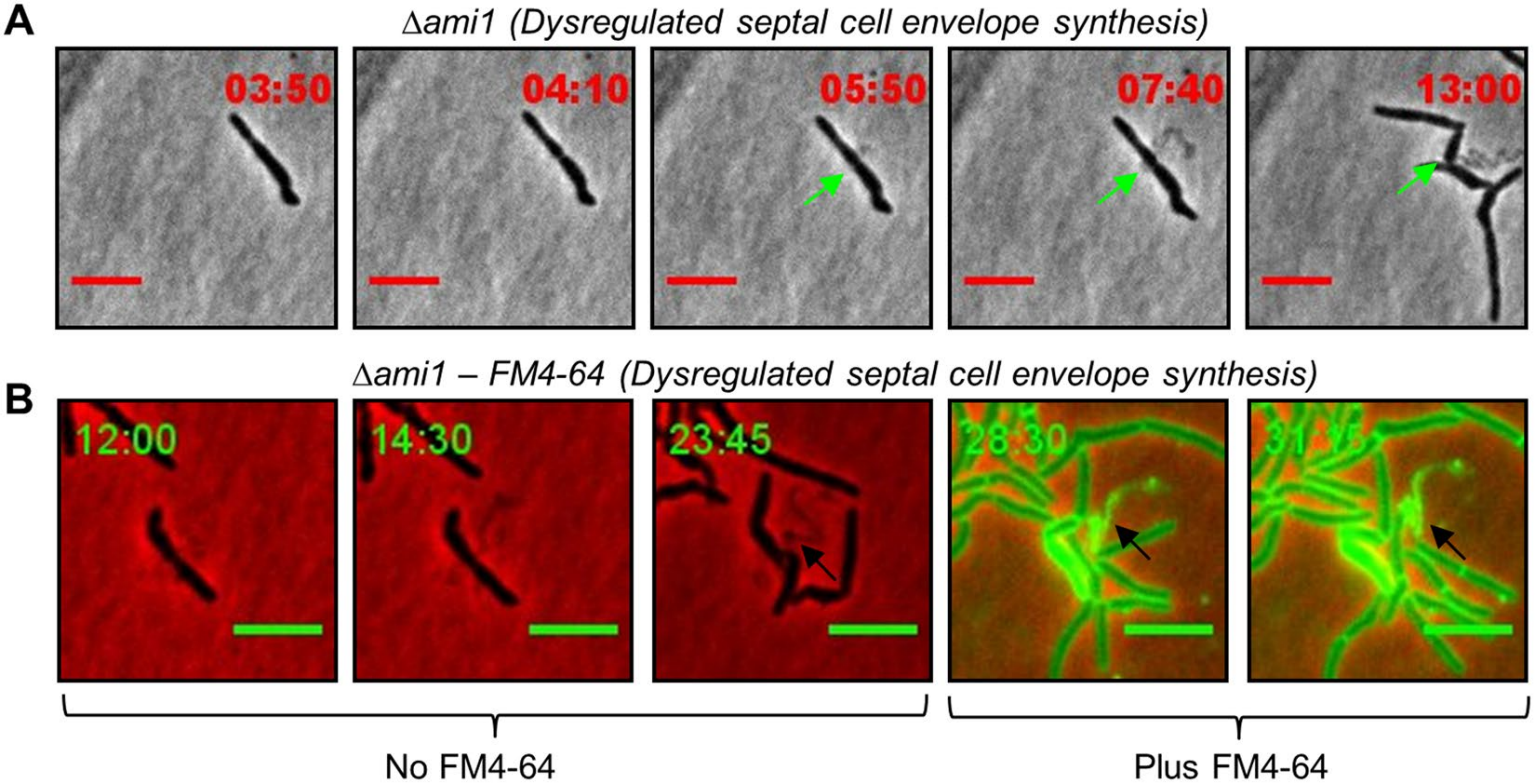
(**A**) Time-lapse microscopic analysis of septal cell wall release in the Δ*ami1* mutant strain. Cells were grown and imaged in Middlebrook 7H9 media. (**A**) Shown is the growth of the Δ*ami1* strain with release of material from the septum (green arrow) suggestive of dysregulated septal envelope synthesis. (**B**) Depicts growth of the Δ*ami1* strain in the presence of FM4-64 (last two frames), the black arrows highlight positive FM4-64 staining of the cell envelope material released from mid-cell. Scale = 5 µm. Time frame is in hours.

Our observations thus far pointed to several defective events occurring at the septa of Ami1 defective cells which, together with the occurrence of abnormal septa, prompted us to carefully examine cell separation events by time-lapse microscopy. We noted that in numerous cases, the failure of cells to separate in the Δ*ami1* mutant resulted in the release of material from the septum, which in some cases remained attached to the newly born cell pole, Fig. 6A, Supplementary Movie 8 and Supplementary Fig. 8 for additional examples. To further probe if the material released was that of the cell envelope, we stained cells with FM4-64 during time-lapse analysis and the strong uptake of this stain confirmed that the material released from the septum was indeed cell wall in composition, Fig. 6B, Supplementary Movie 9. These observations suggested an accumulation of excess septal cell wall that was not coordinately degraded in the absence of Ami1.

### Accumulation of 3-3 crosslinks in the Δ*ami1* mutant

**Figure 7:**
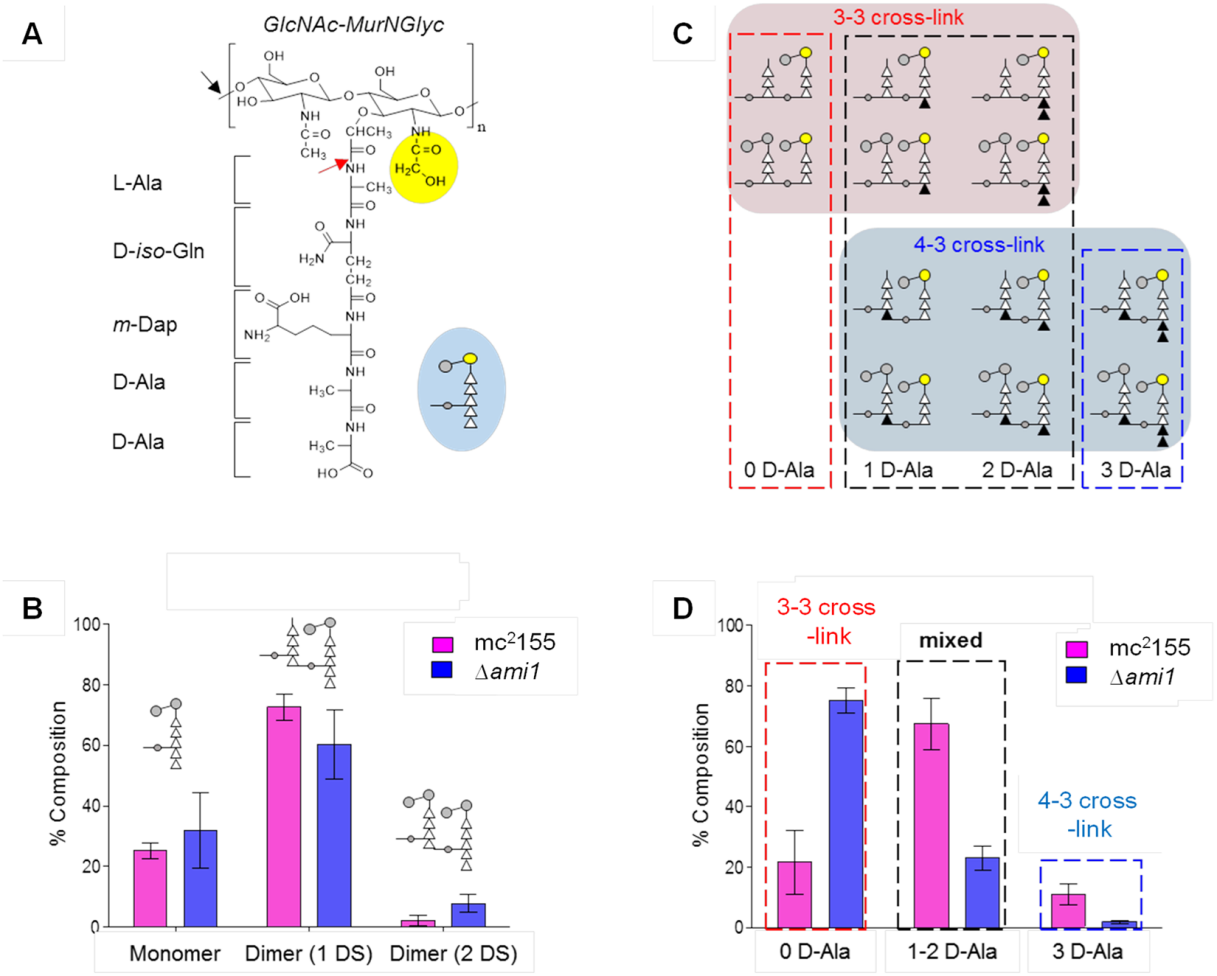
Compositional analysis of peptidoglycan from mc^2^155 and Δ*ami1* mutant strains by LC-MS. (**A**) Chemical structure of the PG-repeat unit in *M. smegmatis*. The disaccharide, GlcNAc-MurNAc, is represented by grey circles, and glycosylated MurNAc as a yellow circle. A pentapeptide stem unit having a sequence L-Ala-D-iso-Gln-*m*-Dap-D-Ala-D-Ala is attached to the disaccharide. The side chain of *m*-Dap, which forms a cross-link with carbonyl carbon of D-Ala in the fourth position from neighbouring repeat unit stem, is shown as a small grey circle. Mutanolysin and the amidases cleave sites indicated by black and red arrows respectively. The blue inset shows the schematic representation of a muropeptide. (**B**) All identified muropeptide species from mutanolysin-digested PG of *M. smegmatis*, independent of glycolylation, amidation, PG-stem modifications and cross-linking types, were classified based on the number of repeat disaccharide units. The isolated muropeptide fragments from wild type *M. smegmatis* were dominated by PG monomers and dimers, indicating low PG cross-linking, which was further reduced in the Δ*ami1* mutant, as evidenced by increased monomers and a decrease in dimers. (**C**) Schematic representation of the chemical structures of PG dimers with variation in stem peptide length. The total number of D-Ala (black triangle) is used to determine the proportion of 3-3 and 4-3 cross-linked dimers. (**D**) Dimer composition of mc^2^155 and Δ*ami1* mutant strains. In mc^2^155, both 3-3 and 4-3 cross-linked dimers are observed. In contrast, 3-3 cross-linked dimers are predominantly found in Δ*ami1* strain. All errors bars represent 95% confidence interval (n = 3).

Considering the accumulation of cell wall at the septum of Ami1 mutant cells, we hypothesized that the overall PG composition of *M. smegmatis* would be different in the absence of Ami1. To test this, we analysed PG composition of mutanolysin-digested cell walls of *M. smegmatis* mc^2^155 and Δ*ami1* strains using LC-MS. Thirty one unique muropeptides species from mc^2^155 and twenty three from the Δ*ami1* mutant were selected for quantitative analysis. Each muropeptide species was quantified by integrating extracted-ion chromatogram (XIC) of select ions. This enabled accurate identification and quantification of muropeptides, as multiple muropeptides that co-elute during chromatographic separation can be resolved in the mass-charge dimension. The isotopic distribution for each selected ion was generated and abundances corrected using an in-house MATLAB program. The normalized intensity-integral sum of all muropeptide species solely based on the number of PG-repeat units is shown in Fig. 7B. All muropeptide modifications including glycolylation, side-chain amidation, PG-stem modifications and PG cross-linking types were combined for the analysis. Representative mass spectra of PG dimers with varying chemical modifications identified from mc^2^155 are shown in Supplementary Fig. 9. Interestingly, the predominant muropeptide species found in mc^2^155 and Δ*ami1* were PG dimers, with oligomers greater than a dimer being rare. This lack of highly cross-linked species indicated that the PG cross-linking in *M. smegmatis* is low. For the Δ*ami1* mutant, an increased fraction of monomers and decreased fraction of dimers suggested a further reduction in PG cross-linking.

For the wild type, the ratio of PG dimers with one to two disaccharides is 33:1 and this ratio decreased to 8:1 for Δ*ami1* (Fig. 7B). The reduction in proportion of PG dimers with one disaccharide in the Δ*ami1* mutant is consistent with Ami1 displaying L-Alanine amidase activity however, the ratio for Δ*ami1* is not zero, suggesting that there are multiple L-Ala amidases in *M. smegmatis*. Two types of PG cross-links (3-3 and 4-3) are found in the cell walls of *M. smegmatis* and the cross-link composition by these types were determined by summing the integrals of muropeptides based on the total number of D-Ala found in the PG stem structure. Schematic representations of differently cross-linked muropeptides are shown in Fig. 7. A large fraction of muropeptides containing 1 or 2 D-Ala in the cell walls of mc^2^155 indicates the presence of both 3-3 and 4-3 cross-linked muropeptides, Fig. 7D. In contrast, cell walls of the Δ*ami1* mutant accumulated 3-3 cross-linked muropeptides with the near absence of PG dimers with 3 D-Ala.

### Loss of Ami1 leads to increased susceptibility to select cell wall targeting antibiotics and enhanced cell permeability

**Figure 8:**
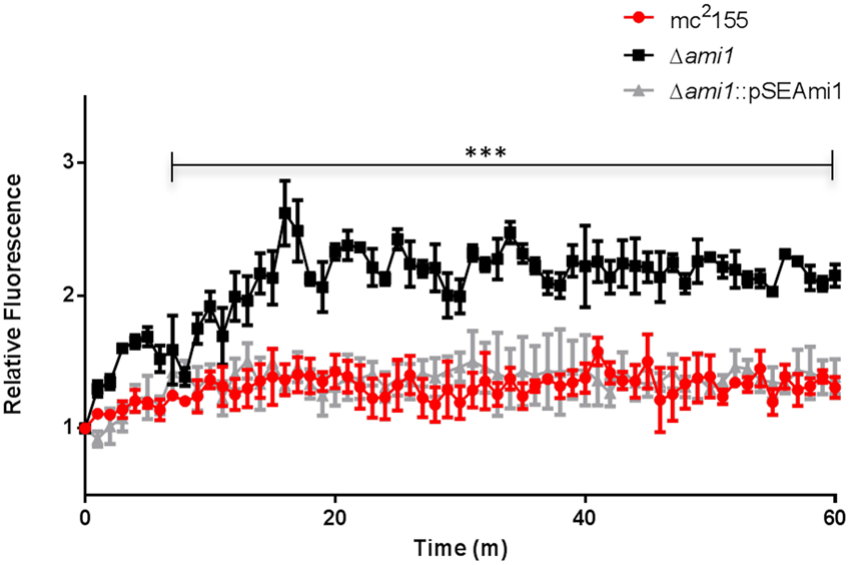
Assessment of cell wall permeability in the Δ*ami1* mutant. Permeability of the mycobacterial cell wall was monitored by quantifying ethidium bromide diffusion into cells over time. The increased fluorescence observed for the Δ*ami1* strain pointed to enhanced ethidium bromide uptake. Data represent three independent experiments where the error bars represented the standard error of the mean. ***p < 0.0001 for both parametric and non-parametric student’s T-test at all points indicated.

We next investigated whether loss of Ami1 affected drug susceptibility of *M. smegmatis*. We assessed the minimum inhibitory concentration of the various classes of antibiotics to the Δ*ami1* mutant and compared them to that of the parental wild type. We found that loss of Ami1 resulted in a four-fold increase in susceptibility to cell wall targeting antibiotics such as vancomycin and various cephalosporins, Table 1. We noted a two-fold increase in susceptibility with numerous drugs tested but did not consider this difference significant and possibly reflective of a growth defect of the Δ*ami1* mutant under the conditions tested. To further investigate these drug susceptibility defects, we tested the permeability of the Δ*ami1* mutant cells using an ethidium bromide diffusion assay and found that the mutant displayed enhanced diffusion of ethidium bromide, Fig. 8.

**Table 1:** Drug susceptibility of the Δ*ami1* mutant.

Drug	WT*	Δami1*^§^	Δ*ami1*::pSEAmi1*
Ampicillin	150	78 (2)	150
Erythromycin	6.25	3.1 (2)	6.25
Vancomycin	0.78	0.20 (4)	0,78125-1,5625
Rifampicin	0.625	0.3125 (2)	0.625
Cefamandole	500	500 (0)	500
Cefoxitin	40	10 (4)	40
Cefotaxime	93.75	23 (4)	93,75-187,5
Ceftriaxone	250	62 (4)	250
Cefapirin	50	25 (2)	50
D-cycloserine	32	62 (-)	32

^*^MIC in µg/mL, ^§^fold increase in susceptibility over wild type is given in parenthesis.

### Cellular Localization of Ami1

**Figure 9:**
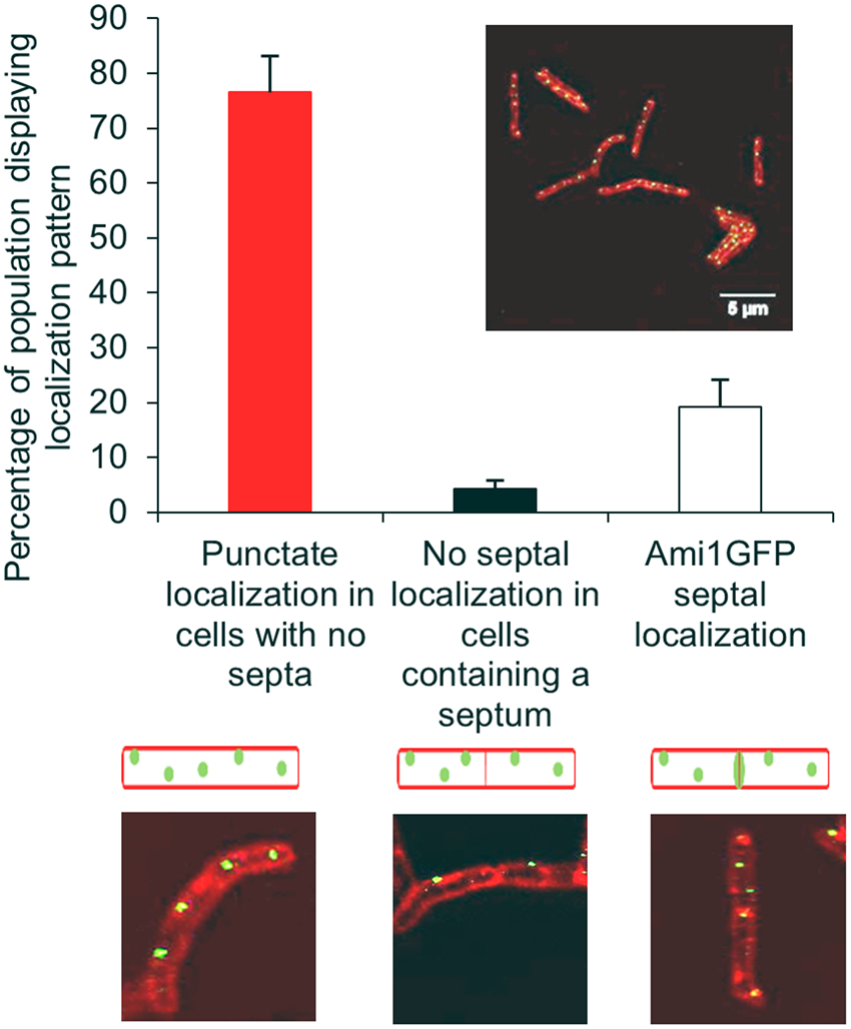
Cellular localization of Ami1-rsEGFP in *M. smegmatis*. Depicted are the three observed localization patterns which include, punctate localization along the lateral axis of the cells with no septa, no septal Ami1 localization in cells containing a septum and septal localization. The histogram depicts the proportion of cells which display the three observed localization patterns. The insert shows a representative view of cells. A total of 589 cells were assessed for localization.

We next sought to determine the cellular localization of Ami1 in *M. smegmatis*. SignalP analysis predicted the presence of a leader-peptide in Ami1, which would allow for translocation to the periplasm, Supplementary Fig. 10. We constructed C-terminal fusions with a green fluorescent protein (rsEGFP), which retains a high fluorescent output. This genetic fusion was expressed under the control of the pmycTET promoter, in the absence of any repressor, to achieve high levels of expression for visualization using bright-field fluorescence microscopy. We were able to test the functionality of the Ami1-rsEGFP fusion derivative by inserting it into the Δ*ami1* strain, followed by microscopic analysis of the strain to determine whether the fusion protein reverted the cell division defects noted with this stain. Complementation of the Δ*ami1* strain with Ami1-rsEGFP abolished all cell division defects, indicating that the fusion protein retained biological activity comparable to the untagged counterpart, Fig. 1C. The Ami1-rsEGFP fusion protein localized in foci along the lateral axis of the cell in a manner that has not been previously reported for any amidase in other bacteria, Fig. 9. While majority of cells imaged display this distribution, we also noted a lower proportion of cells with Ami1-rsEGFP localized at the septum.

## Discussion

Mycobacteria possess a unique cell wall structure and adopt modalities of cell growth and division that are distinct from that reported in well-characterised model organisms such as *E. coli* and *B. subtilis*. As such, the biological roles of many PG hydrolytic enzymes, which are widely distributed in bacteria that have PG, are expected to be divergent to those previously described. In this study, we sought to determine the role of Ami1 in mycobacterial growth and cell division. Numerous studies in both rod-shaped organisms and cocci have identified an important role for cell wall amidases in bacterial cell division, as illustrated by the inability of amidase defective mutants to degrade the septum, resulting in the formation of chains consisting of multiple cells. In this regard, deletion of *amiA*, *amiB* and *amiC* in *E. coli* resulted in the formation of chains consisting of 6 to 20 cells^14^. Deletion of the *lytC*-encoded amidase in combination with a lytic transglycosylase from *B. subtilis* (which generally forms chains) resulted in even longer chains, however LytC appears to play a minor role during cell division as deletion in the wild type background resulted in no septation defects^19^. In *M. smegmatis*, we found that deletion of Ami1 led to severe cell separation defects and the formation of chains of cells connected by unresolved septa. As in *E. coli*, this defect was not lethal but surprisingly, did not lead to a notable defect in growth rates or visible clumping in liquid cultures. Using single-cell time-lapse microscopy we demonstrated that cell growth continues in the Δ*ami1* mutant strain through atypical modalities of cell extension such as lateral budding and Y-form cell growth and division. Lateral buds often proceed to elongate and in many cases, these defective growth processes result in the formation of viable daughter cells. In Δ*ami1* bacterial chains, we noted no anomalies in the uptake of the DAPI stain (data not shown), suggesting that chromosome segregation is unaffected by the failure to degrade the septum. The striking defects in cell separation that occur upon deletion of a single amidase in *M. smegmatis* are unusual and inconsistent with reports of amidase function in other organisms, where deletion of multiple amidases-like genes is required to observe a chaining defect^14, 18^. In this regard, our observations are akin to the deletion of a single amidase in *Neisseria gonorrhoeae*, where deletion resulted in the formation of bacterial masses consisting of multiple cells attached to each other by unresolved cell wall^39^.

In *E. coli*, cell separation amidases localize to the septum, in some cases with activator partnering proteins^21, 40, 41^. Our cellular localization studies with Ami1-rsEGFP revealed that Ami1 is distributed along the lateral axis of the cell and is also found at the septum of dividing cells. This unusual pattern of amidase distribution in the cell has not been reported in other organisms. As deletion of Ami1 leads to branching of cells, we postulate that the localization of Ami1 along the lateral axis of this cell serves as a mechanism to degrade any aberrant branching in wild type bacteria. However, this explanation is largely speculative and requires further studies. Septal localization was observed in some cells that contained septa, suggesting that Ami1 is recruited to the septum at later stages of cell division, purely as a septal hydrolysing enzyme. This observation could not be confirmed with time-lapse microscopy as Ami1-rsEGFP fusion protein was short-lived and could not be easily detected in real-time. Loss of Ami1 also resulted in the occurrence of malformed septa, most likely due to excessive septal PG synthesis that is uncoupled from septal hydrolysis. The release of septal cell wall material in some cases supports this hypothesis and further indicates a lack of co-ordination between the two processes in the absence of Ami1.

We sought to further resolve the cell separation defect in the Δ*ami1* mutant by studying the behavior of the essential cell division protein, FtsZ. We noted the coordinated appearance of two FtsZ rings in the wild type, which eventually coalesced to form a bundled FtsZ polymer with contractile force to effect cell separation. This did not occur in the Δ*ami1* mutant where single rings were observed, which were less bright and diffused compared to wild type and often uncoupled, most likely due to the inability of the cell to degrade the septum. These data confirm that the contractile force generated by the FtsZ ring in mycobacteria needs to be carefully coordinated with septum degradation for successful cell separation. Moreover, the lack of coordinated appearance of two FtsZ rings in the Δ*ami1* mutant suggests that septal degradation is linked to the spatial and temporal placement of FtsZ rings in *M. smegmatis*. In support of this, septal recruitment of AmiC in *E. coli* is dependent on interactions with FtsN, while AmiA septal recruitment and activity requires FtsEX, through interactions with EnvC^21, 22, 41^.

**Figure 10:**
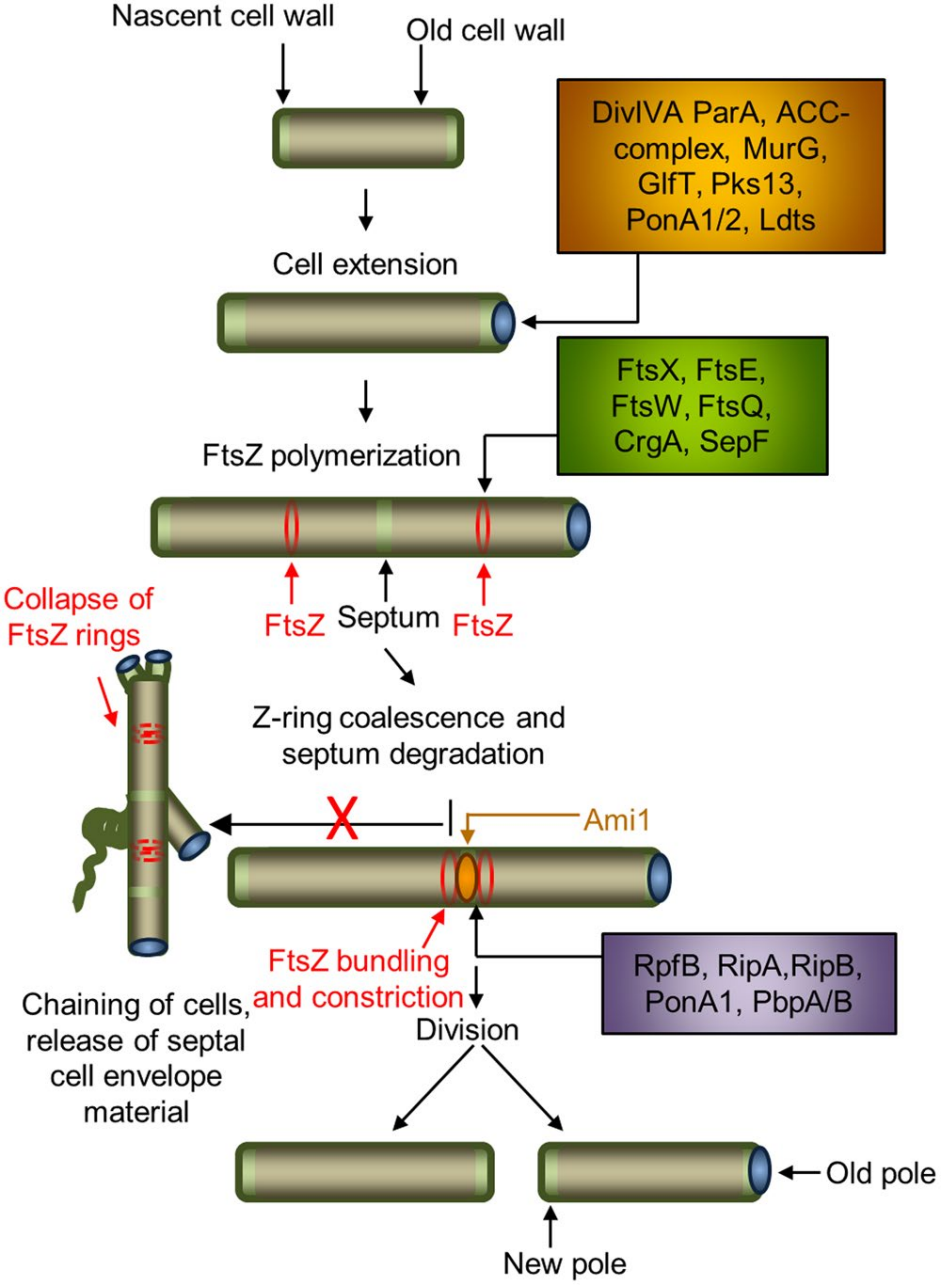
Proposed model for amidase function in mycobacteria. Mycobacterial cell division requires remodelling of the cell wall in two distinct regions namely the polar annular region and the septum. Shown are proteins associated with cell surface restructuring at the pole (orange box) and the septum (purple box). Proteins involved in FtsZ landing, polymerization and bundling are shown in the green box. Ami1 is required for septal cleavage. Deletion of *ami1* results in the formation of cell chains, which are thinner than wild type bacteria, with aberrant cell growth/division modalities such as Y-form cell growth and lateral budding. Ectopic polar growth and lateral budding is mediated by mislocalization of the cell extension machinery. Defective division in cell chains also results in destabilization of FtsZ polymers and dysregulation of septal cell wall synthesis.

Previous work identified a septal degradation complex in *M. smegmatis* that comprises RpfB, RipA and PonA1 (PBP1) wherein RpfB is required for synergistic PG septal degradation by the endopeptidase, RipA^11^. This complex is antagonistically regulated by the penicillin binding protein 1 (PonA1)^12^ and depletion of these essential proteins results in cell separation defects. Considering the prevailing evidence, the presence of similar defects in the Δ*ami1* mutant points to two possible cell separation modalities in *M. smegmatis*, as the presence of the RpfB-RipA-PonA1 complex was not able to compensate completely for loss of *ami1* in the majority of cells assessed. However, the presence of Δ*ami1* cells that were able to correctly coordinate FtsZ and DivIVA localization to allow for cell separation suggests that the RpfB-RipA-PonA1 complex is sufficient in some cells to allow for cell division in the absence of Ami1. This stochastic nature of this compensatory mechanism could be related to the way in which these different proteins interact with other components of the divisome or may be associated with specific temporal events during septal degradation. Furthermore, these enzymes cleave distinct bonds in the PG and it may be possible that the inability to degrade either the bond between the glycan and the stem peptide (by amidases) or the peptide bond between positions 2 and 3 in the stem peptide (by RipA) leads to defective cell division, Fig. 10.

Several studies have demonstrated that mycobacterial PG is composed of 3-3 and 4-3 crosslinks, the former being produced through the activity of L,D-Transpeptidases. However, the functional consequences of these PG modifications remain unknown. Our analysis identified an accumulation of 3-3 crosslinked muropeptides in the Δ*ami1* mutant. Given that there is an increase in septal PG in this strain, this observation suggests that mycobacterial septal PG is predominantly in the 3-3 crosslinked conformation. The dysregulation of other forms of cross-linked PG in the Δ*ami1* mutant also suggests that failure to carefully coordinate synthesis and degradation of septal PG leads to global changes in PG composition and stability, the latter evidenced by the increased sensitivity of the Δ*ami1* mutant to PG targeting antibiotics and enhanced permeability of Δ*ami1* mutant cells.

One of the more unexpected observations was the formation of lateral branches. Unlike other actinomycetes such as *Streptomyces*, mycobacteria do not grow by lateral budding. To understand how loss of Ami1 resulted in these modalities of growth, we analysed the behaviour of DivIVA and noted two scenarios: (I) Septal branching as a product of stalled septum cleavage, resulting in the recruitment of DivIVA at the septum, leading to the formation of a pole and thereafter, the lateral bud at the septum or (II) Lateral branching at a non-septal region as a result of ectopic DivIVA placement along the lateral axis of the cell during elongation. Mycobacterial growth and division are uncoupled events and the organism continues to grow regardless of the occurrence of cell division^37, 42^. It is plausible to speculate that a stalled septum forces the elongasome to rearrange to form a lateral bud for survival of the cell.

Collectively our study describes the function of a cell division amidase and to our knowledge, represents the first extensive biological characterisation of this enzyme in mycobacteria. Concerning the remaining mycobacterial amidases, CwlM has been shown to play an essential role in peptidoglycan synthesis through the activation of MurA^31^. Ami3 has not been investigated and the physiological role of this enzyme in bacterial growth is unknown. The substitution of essential amino acids in Ami3 suggests possible loss of function or altered catalytic activity. Saturating transposon mutagenesis conducted to identify genes required for mycobacterial tolerance against oxidative damage identified Ami4 as one of the crucial enzymes in protecting the bacterium against reactive oxygen intermediates^43^. In this regard, it is worth noting that no other cell wall hydrolysing enzyme was identified in this analysis. A similar function for amidases has been observed in *Cyanobacterium Anabaena* PCC 7120, where AmiC1 was shown to play an important role in the formation of heterocysts which are specialized cells that protect the cytoplasmic components against O_2_^44^. This points to the possibility that although these enzymes essentially catalyse the same reaction, they may play non-redundant, essential roles in bacterial growth and survival.

## Methods

### Bacterial strains, plasmids and growth conditions

All bacterial strains and plasmids created and/or used in this study are listed in Supplementary Table 1 and Supplementary Table 2 respectively. *E. coli* strains were grown in Luria-Bertani broth (LB) at 37 °C with shaking at 100 rpm or Luria-Bertani Agar (LA). Antibiotic supplementation was done at the following concentrations: Kanamycin (Kan) 50 μg/mL, Hygromycin (Hyg) 200 μg/mL and Ampicilin (Amp) 100 μg/mL. *M. smegmatis* strains were grown in Middlebrook 7H9 liquid media supplemented with 0.2% glucose, 0.5% glycerol, 0.085% NaCl, 0.05% Tween 80 and appropriate antibiotic at 37 °C (herein referred to as 7H9 media) with shaking at 100 rpm unless stated otherwise. Antibiotic concentrations used were as follows: Kan 25 μg/mL and Hyg 50 μg/mL. Strains were also grown on Middlebrook 7H10 solid media supplemented with 0.2% glucose, 0.5% glycerol, 0.085% NaCl and appropriate antibiotics at 37 °C unless stated otherwise (herein referred to as 7H10 media).﻿ To generate a deletion mutant of Ami1, primers shown in Supplementary Table 3 were designed to amplify the upstream and downstream regions of homology for the *ami1* gene, which were then fused to yield an out-of-frame deletion allele. The mutant allele was cloned into p2NIL (Supplementary Table 2), followed by cloning of a marker gene cassette. The resulting suicide substrate was electroporated into wild type *M. smegmatis* mc^2^155 and subsequent two step allelic exchange mutagenesis yielded the Δ*ami1* mutant.﻿

### Bioinformatics analysis

Bioinformatics analysis was carried out using a number of online tools and databases. *E.coli* DNA and protein sequences were downloaded from the Biocyc *E. coli* database (http://www.biocyc.com/ecocyc/index.shtml). *M. smegmatis* DNA and protein sequences were downloaded from the Ecole Polytechnique Fédérale de Lausanne Smegmalist database (http://mycobrowser.epfl.ch/smegmalist.html) and *M. tuberculosis* DNA and protein sequences were downloaded from the Ecole Polytechnique Fédérale de Lausanne Tuberculist database (http://tuberculist.epfl.ch/). Protein and DNA sequence BLAST searches were conducted using the NCBI Blast tool (http://blast.ncbi.nlm.nih.gov/Blast.cgi), Protein domain determination was conducted using the Sanger institute pfam tool (http://pfam.sanger.ac.uk/), determination of signal peptides was conducted using the SignalP version 4.1 online tool (http://www.cbs.dtu.dk/services/SignalP/) and protein alignment was conducted using the EMBL-EBI clustalW tool (http://www.ebi.ac.uk/Tools/msa/clustalw2/).

### Phenotypic analysis

Growth rates of wild type and mutant strains were determined with growth curves, carried out by inoculating either 1 mL of frozen culture or a single colony into 15 mL 7H9 containing appropriate antibiotics and incubated overnight at 37 °C shaking at 100 rpm. The preculture was then diluted to a final optical density (OD)_600nm_ of 0.05 in 25 mL 7H9 containing appropriate antibiotic, when necessary, and incubated at 37 °C with shaking at 100 rpm. Growth was determined by recording OD_600nm_ measurements at 3 hour intervals and data was displayed as a line graph. Sliding motility assays were carried out using the protocol described by Martinez *et al*.^45^. Briefly, strains were streaked on 7H10 containing appropriate antibiotics and incubated at 37 °C until single colonies could be observed. A single colony was spotted on M63 minimal media [100 mM KH_2_PO_4_, 15 mM (NH_4_)_2 s_O_4_, 1.7 µM FeSO_4_.7H_2_O, pH to 7.0 with KOH containing 0.3% agar with 0.5% glycerol as a carbon source]. The plates were sealed with parafilm and incubated at 37 °C for two weeks and images were taken using the G-Box SYNGENE system. Spotting assays were conducted to observe the morphologies of bacterial strains when grown on solid media. Cultures were grown in 7H9 containing appropriate antibiotics to an OD_600nm_ = 0.8. A ten-fold serial dilution series from 10^0^–10^6^ was made and 10 µl of each dilution was spotted on 7H10 media containing appropriate antibiotic and incubated at 37 °C.

### Scanning electron microscopy

For cell preparation, 50 mL cultures were grown in 7H9 containing appropriate antibiotic to an OD_600nm_ = 0.8. The cells were harvested by centrifugation, washed twice with PBS and resuspended in 2.5% glutaraldehyde in PBS overnight at 4 °C. The cells were then washed twice with PBS and resuspended in 100 µl of 2% osmium tetraoxide in PBS and incubated at room temperature for 1 hour, followed by dehydration in a series of ethanol concentrations for 2 min at each concentration beginning with 30% then 50%, 70% and twice at 100% ethanol followed by storage in 100% ethanol. Cells were spotted on a filter, coated twice with carbon and viewed using the FEI Nova NenoSEM 230. Electron micrographs were manipulated through in silico magnification of specific regions or for brightness/contrast. In the case of brightness and contrast, any manipulations were applied to the entire image.

### Transmission electron microscopy

A 50 mL culture was grown in 7H9 broth containing appropriate antibiotic to an OD_600nm_ = 0.8. The cells were harvested by centrifugation, washed twice in 1 mL PBS and resuspended in fixing solution containing 0.1 mM HEPES, 2% (v/v) formaldehyde, 2.5% (v/v) glutaraldehyde and 0.05% (w/v) Ruthenuim red, and incubated at room temperature for 1 hour. The cells were then harvested and resuspended in 100 µl of 2% osmium tetraoxide in PBS and incubated at room temperature for one hour. The samples were dehydrated as described above. The dehydrated samples were then washed in propylene oxide twice and incubated for one hour in a solution made up of 50% propylene oxide and 50% resin mixture which consists of 5.62 g araldite, 7.75 g epon 812 and 15 g Dodecenyl succinic anhydride. The propylene oxide resin mixture was then removed and the cells were resuspended in 100% resin mixture and incubated at room temperature overnight. Cells were pelleted, the resin mixture was removed and fresh resin mixture containing Dimethylaminomethyl phenol (DMP) 30 at a ratio of 1:40 of DMP 30 to resin mixture was added to the cells and these were incubated at 60 °C for 48 hours. The solidified resin was sectioned using a Relchert Ultracut Ultramucrotome (Circa 1980) and viewed using a Tecnai F20 TEM. Electron micrographs were manipulated through in silico magnification of specific regions or for brightness/contrast. In the case of brightness and contrast, any manipulations were applied to the entire image.

### Construction of fluorescently tagged strains

Vectors for expressing fluorescently tagged proteins were constructed as follows: *ami1, ftsZ* and *rsEGFP* genes were amplified using the primer sets outlined in Supplementary Table 3. The amplified products were restricted at the engineered restriction site, resulting in a *Sph*I-*Eco*RI (*ami1*) fragment, an *Xba*I-*Eco*RI (*ftsZ*) fragment and an *Eco*RI*-Pst*I (*rsEGFP*) fragment. The *ami1* gene was cloned with *rsEGFP* into pSE100, downstream of the *tet* operator, and *ftsZ* was cloned with rsEGFP into pMV306(H). The resulting plasmids were electroporated into *M. smegmatis*.

### Timelapse microscopy

The different mycobacterial strains were grown in microfluidic devices and time-lapse microscopy experiments carried out as described before^42^. Briefly, the bacterial cultures were grown to exponential phase in ink-well bottles at 37 °C. Clumps of bacteria were removed by concentrating the cells 10-fold and filtering through a 5 μm filter. The bacteria were seeded into a microfluidic device and imaged on a Delta vision Personal DV imaging system (GE HealthCare Life Sciences) using a 100x objective (Olympus Plan Semi Apochromat, 1.3 NA). The images were acquired at 10 or 15 min intervals using a CoolSnap HQ2 camera. Images were acquired on FITC (GFP fluorescent reporter strains; excitation 490/20; emission 528/38); TRITC/Cy5 (FM4-64 staining; excitation 542/27; emission 676/34). Middlebrook 7H9 medium with or without 0.2 μg/mL FM4-64 (ThermoFisher Scientific) was circulated through the device at a flow rate of 25 μl/min. Images were analysed and processed using Softworx 4.1 (Applied Precision, GE HealthCare) or ImageJ v 1.47n (Reference - Rasband, W.S., ImageJ, U. S. National Institutes of Health, Bethesda, Maryland, USA, http://imagej.nih.gov/ij/, 1997–2015). All experiments were repeated at least two times.

### Isolated and digestion of cell walls for LC-MS

Pre-cultures for each strain were prepared by inoculating single colonies into 5 mL 7H9 supplemented with glucose salts, Tween 80 and antibiotics where appropriate and incubated at 37 °C for 24 hrs. The entire volume was subsequently used to inoculate 500 mL of culture media (as above) which was incubated for two days. Bacterial cells were harvested at 3082 × *g* for 20 min and the supernatant was discarded. Pellets were re-suspended in 6 mL 1 × PBS (0.2 µm filter-sterilized) and cells were lysed using a MagNA lyser (Roche) with the following settings: three 40 s cycles at 5500 rpm with incubation on ice for 2 min between cycles. Cell wall fragments were collected by centrifugation at 15000 *g* for 15 min at 4 °C. Pellets were re-suspended in 1 mL boiling 1 × PBS with 8% SDS, boiled for a further 30 min and then cooled to room temperature overnight. Cell wall material was collected by centrifugation at 20000 *g* for 30 min at 4 °C and washed thrice with sterile water (using the same centrifugation parameters between washes). Pellets were re-suspended in 1 mL Tris-Cl (pH 7.4) and digested with 0.3 mg (30 µl of 10 mg/mL stock) of pronase at 37 °C for 24 hours. Pronase-digested cell wall material was then collected by centrifugation at 20000 × *g* for 30 min at 20 °C and washed thrice with distilled water. Cell wall material was subsequently re-suspended in 1 mL 2 × PBS (pH 7.4) and digested with 0.2 mg (20 µl of 10 mg/mL stock) at 37 °C for 24 hrs. Digested material was harvested at 20000 × *g* for 3 min at 20 °C, washed thrice with distilled water and stored at −20 °C before proceeding to further enzymatic digestions. Isolated cell walls were digested by mutanolysin (Sigma-Aldrich) with the addition of 0.66 KU at room temperature for 24 hours, followed by repeated addition of 0.66 KU mutanolysin to the mixture for 24 additional hours. The samples were then frozen and lyophilized. Lyophilized muropeptides were dissolved in 1 mL of 0.375 M sodium borate buffer (pH 9.0) and reduced by addition of 10 mg of sodium borohydride in 960 µL of borate buffer at room temperature for 30 min. The reduction was quenched by addition of 125 µL of 85% phosphoric acid, frozen at −80 °C, and then lyophilized.

### Liquid chromatography-mass spectrometry

Mutanolysin-digested cell walls in 500 μL of 20 mM Tris pH 8.0 buffer were diluted 1:10 in methanol with 0.1% formic acid. A Waters Synapt G2 High Definition Mass Spectrometer (HDMS) Time-of-Flight (TOF) mass analyzer coupled with Waters C18 ACUITY Ultra Performance Liquid Chromatography (UPLC) was used to analyze mutanolysin-digested muropeptide fragments. Chromatographic separation of mutanolysin-digested PG was carried out by injecting 1 μL of the sample from a 5 μL sample loop to the column under isocratic condition of 99% buffer A (99.8% anhydrous methanol with 0.1% formic acid) and 1% buffer B (100% acetonitrile) for 5 minutes. A Waters nanoACQUITY C18 reverse-phase column (75 μm × 100 mm, packed with 1.7 μm beads with 130 Å pore size) with nanoACQUITY C18 trap column (180 μm × 20 mm, 5 μm beads with 100 Å pore size) was used for chromatographic separation. First, a linear gradient to 50% buffer B was applied for 60 minutes. The column was subsequently regenerated under isocratic condition with 85% buffer B for 5 minutes, a linear gradient to 98% buffer A for 1 minute, then isocratic at 98% buffer A for 23 minutes. The flow rate was kept constant (0.6 μL/min) throughout the analysis. The sample was ionized by nanoflow electrospray ionization with spray voltage of 35 V and capillary voltage of 3.5 kV. The Synapt G2 HDMS with TOF mass analyzer (Waters) was run in positive ion mode. Fibrinopeptide B was used as an internal standard to correct for the drift of the instrument. Data were analyzed using MassLynx (Waters) and MATLAB (MathWorks).

### Fluorescence microscopy

Cells were grown in 10 mL 7H9 broth containing appropriate antibiotics to an OD_600nm_ = 0.8 followed by harvesting and washing twice in cold HANK’S buffered salt solution. Cells were centrifuged, resuspended in a 5 µg/mL FM4-64 (Invitrogen) staining solution and placed on ice for 10 minutes, harvested, resuspended in HANK’S buffered salt solution and spotted on agar pads. Images were then taken using a 100×, 1.46 numerical aperture objective mounted to an Axio Observer Z1 base (Zeiss). Images were taken using an AxioCam HRm camera and processed using the Zen blue Ver 5.1.2600 (Zeiss). Images were manipulated for brightness and contrast using the ImageJ V 1.8 to allow for clear visualization of specific dyes. These manipulations were applied to the entire image. In some cases representative bacteria are shown for quantification purposes. For protein localization studies, a custom-built TIRF-SIM system was used as previously reported^46^. The key component of TIRF-SIM system was a spatial light modulator (SLM) that functioned as a programmable phase grating. The period and orientation of the phase grating was adjusted with sub-millisecond switching times. For two-colour TIRF-SIM images, the raw images from the green and red channels were captured by separate sCMOS cameras (Hamamatsu, Orca Flash 4.0 v2 sCMOS) with 512 × 512 pixels for the region of interest area. The exposure time of each raw image was set to 30 ms, so the total acquisition time for two channels was ca. 0.6 sec. High-resolution images were reconstructed with an algorithm described previously. The resolutions of reconstructed green and red channel images are 97 and 112 nm, respectively.

### Ethidium bromide diffusion assay to monitor cell permeability

*M. smegmatis* was grown in 20 mL of 7H9 medium at 37 °C to an OD_600nm_ = 0.8. Cultures were centrifuged at 4500 rpm for 10 minutes, the supernatant discarded and the pellet washed in PBS. Thereafter, the pellet was resuspended in PBS containing 0.4% glucose then the OD_600nm_ was adjusted to 0.4. A 95 µl aliquot of bacterial suspension was placed into flat bottom, black 96-well plates, followed by addition of ethidium bromide to final concentration of 8 µg/mL. Fluorescence was measured with an excitation and emission wavelength of 530 nm and 585 nm respectively, every 60 seconds for 60 minutes at 37 °C.

## Electronic supplementary material

Supplementary Information

Supplementary Movie S1. Single-cell time-lapse microscopy of wild type M. smegmatis mc2155.

Supplementary Movie S2. Single-cell time-lapse microscopy of the M. smegmatis Δami1 mutant.

Supplementary Movie S3. Single-cell time-lapse microscopy of DivIVA localization in wild type M. smegmatis mc2155.

Supplementary Movie S4. Single-cell time-lapse microscopy of DivIVA localization, in the septal region, of the M. smegmatis Δami1 mutant.

Supplementary Movie S5. Single-cell time-lapse microscopy of DivIVA, along the lateral axis of the cell, in the M. smegmatis Δami1 mutant.

Supplementary Movie S6. Single-cell time-lapse microscopy of FtsZ localization in wild type M. smegmatis mc2155.

Supplementary Movie S7. Single-cell time-lapse microscopy of FtsZ localization in the M. smegmatis Δami1 mutant.

Supplementary Movie S8. Single-cell time-lapse microscopy of the M. smegmatis Δami1 mutant depicting release of material from the septum.

Supplementary Movie S9. Single-cell time-lapse microscopy of septal cell wall release in the M. smegmatis Δami1 mutant.
