# Supplementary material for: An Amidase_3 domain-containing N-acetylmuramyl-L-alanine amidase is required for mycobacterial cell division
Source: Sci Rep. 2017 Apr 25;7:1140. doi: 10.1038/s41598-017-01184-7 (PMC5430687; doi:10.1038/s41598-017-01184-7)
Supplement: Supplementary file 1 — Supplementary Information [file 41598_2017_1184_MOESM1_ESM.pdf]

An Amidase_3 domain-containing *N*-acetylmuramyl-L-alanine amidase is required for mycobacterial cell division

Supplementary Information

Sibusiso Senzani[1], Dong Li[2], Ashima Bhaskar[3], Christopher Ealand[1], James Chang[4], Binayak Rimal[5], Chengyin Liu[4], Sung Joon Kim[4], Neeraj Dhar[3] and Bavesh Kana[1]

[1]DST/NRF Centre of Excellence for Biomedical TB Research, Faculty of Health Sciences, University of the Witwatersrand, National Health Laboratory Service, Johannesburg, 2001, South Africa; [2]Howard Hughes Medical Institute, Janelia Research Campus, Ashburn, Virginia, USA; [3] Global Health Institute, Ecole Polytechnique Fédérale de Lausanne, Lausanne, Switzerland; [4] Baylor University, Department of Chemistry & Biochemistry, Waco, Texas, USA; [5] Baylor University, Institute of Biomedical Studies, Waco, Texas, USA.

Keywords: amidase, cell separation, *Mycobacterium*, peptidoglycan

For Correspondence: Kana, B.D. (bavesh.kana@nhls.ac.za), Telephone: +27 11 489 9030,Fax: +27 11 489 9397. Cnr De Korte and Hospital street, National Health Laboratory Service, James Murray building, room 201, Braamfontein, Johannesburg, 2001

Supplementary Tables

Table S1: Bacterial strains created/used in this study

Strain	Genotype	Reference
Escherichia coli DH5α	*SupE44 ΔlacU169 hsdR17 recA1 endA1 gyrA96 thi-1 relA1*	Promega, Madison, WI
mc^2155	High frequency transformation mutant of *M. smegmatis* ATCC 607	[1]
Δ*ami1*	Derivative of mc^2155 carrying an unmarked, out-of-frame deletion in *ami1*	This study
Δ*ami1*::pSEami1	Derivative of Δ*ami1* carrying pSEami1 integrated at the bacterial *attB* phage attachment site, Hyg^R	This study
mc^2155 (pAmi1GFP)	Derivative of mc^2155 carrying an episomal pSAmi1GFP vector, Hyg^R	This study
mc^2155 (pDivIVAGFP)	Derivative of mc^2155 carrying an episomal plS220 vector, Hyg^R	This study
Δ*ami1* (pDivIVAGFP)	Derivative of Δ*ami1* carrying an episomal plS220 vector, Hyg^R	This study
mc^2155::pFtsZGFP	Derivative of mc^2155 carrying pMFtsZGFP intergrated at the *attB* bacterial phage attachment site, Hyg^R	This study
Δ*ami1*::pFtsZGFP	Derivative of Δ*ami1* carrying pMFtsZGFP intergrated at the *attB* bacterial phage attachment site, Hyg^R	This study

Kan^R: Kanamycin Resistance, Hyg^R: Hygromycin Resistance

Table S2: Plasmids created and/or used in this study

Plasmids	Genotype	Reference
p2NIL	*E. coli* cloning vector; Kan^R	[2]
pSE100	*E. coli*-Mycobacterium shuttle vector carrying P_{myc1} *tetO*; Hyg^R	[3]
pGEM3Z(+)f	*E. coli* cloning vector; Amp^R	Promega
pGOAL19	Plasmid carrying *lacZ*, *sacB* and *hyg* genes as a *Pac*I cassette; Amp^R	[2]
pMV306(H)	pMV306 derivative carrying Hyg^R marker	H. Boshoff
pGEMAmi1US	Derivative of pGEM3Z (+)f carrying 1704 bp upstream homologous region of *ami1*, containing 1655 bp upstream of *ami1* and 31 bp of the 3' end of *ami1*; Amp^R	This study
pGEMAmi1DS	Derivative of pGEM3Z (+)f carrying 1674 bp downstream homologous region of *ami1*, containing 1626 bp downstream of *ami1* and 13 bp of the 3' end of *ami1*; Amp^R	This study
p2NILΔAmi1	Derivative of p2NIL carrying the Δ*ami1* deletion allele, with a 751 bp internal deletion; Kan^R	This study
p2ΔAmi1G19	Derivative of p2NILΔAmi1 carrying the *lacZ*, *sacB* and *hyg* genes from pGOAL19; Hyg^R, Kan^R	This study
pSEAmi1	Derivative of pSE100(H) carrying a functional *ami1* gene and the upstream native promoter region Hyg^R	This study
pAmi1GFP	Derivative of pSE100 carrying the *ami1-rsEGFP* fusion gene downstream the tetO Hyg^R	This study
plS220	Derivative of pMV361 carrying the *wag31-GFP* fusion gene downstream the *hsp60* Kan^R	[4]
pFtsZGFP	Derivative of pMV306(H) carrying the *ftsZ-rsEGFP* fusion gene downstream the *ftsZ* native promoter Hyg^R	This study

Kan^R: Kanamycin Resistance, Hyg^R: Hygromycin Resistance, Amp^R: Ampicillin Resistance

Table S3: All Primers used in the study

Name	Primers	Product properties
**Primers for mutant construction**		
Ami1USF	GCCGCCGGATCCCAGGTACAGCCCGATGCT	1704 bp amplicon including 31 bp of the 5' end region of the *ami1* gene and full length
Ami1USR	GCCGCCGAATTCGCAGACAGGCTGGGACTC	MSMEG_6280 and *recR* genes
Ami1DSF	GCCGCCGAATTCTGCGTTGACCCTCGGCTA	1674 bp amplicon including 13 bp of the 3' end region of the *ami1* gene, a full length
Ami1DSR	GCCGCCAAGCTTCGCTGTCACGCAACCGTAG	MSMEG_6282 genes and 1126 bp of MSMEG_6283
**Primers for screening of mutant genotypes or plasmid integration**		
Ami1DelScrF	TCTCGGTTGCGTCTTCTTG	*ami1* internal revese primer producing 608 bp wild type *ami1* amplicon
Ami1DelScrR1	CGTCCCTGGTGAGCAACT	
Ami1DelScrR2	ACGAGTACAGCACGTGGAA	Second reverse primer for *ami1* producing 327 bp Δ*ami1* amplicon
AttBS1	ACGTGGCGGTCCCTACCG	282 bp fragment indicating pMV vector
AttL2	CTTGGATCCTCCCGCTGCGC	intergration into tRNA glycin *attB* site
AttBS2	ACAGGATTTGAACCTGCGGC	320 bp fragment indicating pMV vector
AttL4	AATTCTTGCAGACCCCTGGA	intergration into tRNA glycin *attB* site
**Primers for cloning the ami1 gene for complementation**		
Ami1CompF	GCGCGCTCTAGACTGCGCCATGATCGTCAC	1203 bp DNA fragment containing the 400 bp region upstream of the *ami1* gene and the full
Ami1CompR	GCCGCCAAGCTTGGAGAACCTCAAGAAGCA	length *ami1* gene
**Primers for construction of fluorescent derivatives**		
Ami1FloF	GCGCCGCCGCATGCCGCGACGCGGCTACCGTG	803 bp fragment containing the full length *ami1* gene
Ami1FloR	GCGCGCGCGAATTCACGCACGGGGCTGACGGC	
FtsZFloF	CGCGCGTCTAGACGCGTCGATCACGTTGAC	1555 bp fragment containing the full length *ftsZ* gene
FtsZFloR	GGCGGCGAATTCGTGCCGCATGAAGGGCGG	
rsEGFPFloF	CGCGCGGAATTCATGGTGAGCAAGGGCGAGGA	792 bp fragment containing the full length *rsEGFP* gene
rsEGFPFloR	GCGCGCCTGCAGAGGAGTCCAAGCTCAGCTAA	

Supplementary Figures

A

```
                                β1       α1   β2     β3                 α2
B. subtilis 168 CwlC            IFIDPGHGGSDPGA-TG--------------------NGLQEKTLTLQIA
B. licherniformis CwlM          IFIDPGHGGSDTGA-SA--------------------NGLQEKQLTLQTA
Paenibacillus polymyxa CwlU     VVIDPGHGGKDPGAGSV--------------------TGRHEKEFTLAVA
E. coli K12 AmiA                VVLDPGHGGIDTGAIGR--------------------NGSKEKHVVLAIA
E. coli K12 AmiC                IMLDPGHGGEDSGAVGK--------------------YKTREKDVVLQIA
E. coli K12 AmiB                IAIDAGHGGQDPGAIGP--------------------GGTREKNVTIAIA
B. subtilis 168 CwlB             IFIDPGHGDQDSGAIG---------------------NGLLEKEVNLDIA
B. anthracis 4229 pXO2-42       IIIDPGHGGIDGGHKG---------------------IYMNESPVVYDTA
M. smegmatis mc2155 Ami2        VIIDPGRGGSDHGLIMQGP-----------------HGPISEADILWDLA
M. tuberculosis H37Rv Ami2      IIIDPGRGGVDHGLIAQGP-----------------AGPISEADLLWDLA
M. leprae TN Ami2               IIIDPGRGGADRGLITQGP-----------------TGPISEADVLWDLA
M. tuberculosis H37Rv Ami1      VFIDPGHNGANDASIGRQVPTGRGGTKNCQASGTSTNSGYPEHTFTWETG
M. leprae TN Ami1               VFIDPGHNGSNDASISRQVPTGRGGTKDCQASGTSTNSGYPEHTFTWDTA
M. smegmatis mc2155 Ami1        VFLDPGHNGANDASIGRQVPTGRGGTKNCQESGTATDDGYPEHSFTWDTT
                                : :*.*:.. : .                            *  .
                                      (H341)                            (E355)

                                   α2          β4                   α3      β5
B. subtilis 168 CwlC            LALRTILTNEYEGVSLLLSRTS------DQYVSLNDRTNAANNWGADFFL
B. licherniformis CwlM          LALRNMLLNEYQNVSVLLSRTS------DQTVSLTQRTNAANSWGADYFL
Paenibacillus polymyxa CwlU     LKVQQLAQNDPD-IQIVLTRNG------DTYPTLDERPQLANNQQASVFV
E. coli K12 AmiA                KNVRSILRNHG--IDARLTRSG------DTFIPLYDRVEIAHKHGADLFM
E. coli K12 AmiC                RRLRSLIEKEGN-MKVYMTRNE------DIFIPLQVRVAKAQKQRADLFV
E. coli K12 AmiB                RKLRTLLNDDPM-FKGVLTRDG------DYFISVMGRSDVARKQNANFLV
B. subtilis 168 CwlB             KRVNTKLNASG--ALPVLSRSN------DTFYSLQERVNKAASAQADLFL
B. anthracis 4229 pXO2-42       VRVQKLFAQKTP-FTALLTRDAYSRPGKNATDSLGKRVEFAKKNKGDIFV
M. smegmatis mc2155 Ami2        SRLEGRMTAIG--MDTVLSRPP------NRSPSDAERAQTANGVGADLMI
M. tuberculosis H37Rv Ami2      SRLEGRMAAIG--METHLSRPT------NRSPSDAERAATANAVGADLMI
M. leprae TN Ami2               SRLEGRMAAIG--IETFLSRTT------NGSPSDSERAAIANNVGAELMI
M. tuberculosis H37Rv Ami1      LRLRAALNALG--VRTALSRGN----DNALGPCVDERANMANALRPNAIV
M. leprae TN Ami1               LQVRAALDGLG--VRTALSRGD----DTGLGPCVDARANMANALHPNASV
M. smegmatis mc2155 Ami1        LRVRAALTALG--VRTAMSRGN----DNALGPCVDERAAMANSLRPHAIV
                                  :.             ::*                *   *        :

                                β5       β6              α4
B. subtilis 168 CwlC            SIHVN----SGGGTGFESYIYPDVG-------------------------
B. licherniformis CwlM          SIHMN----AGGGTGFEDYIYPGVG-------------------------
Paenibacillus polymyxa CwlU     SIHGNSMPTSNNGKANGSETYYAR--------------------------
E. coli K12 AmiA                SIHADGF-TNPKAAGASVFALSNRGASSAMAKYLSERENRADEVAGKKA-
E. coli K12 AmiC                SIHADAF-TSRQPSGSSVFALSTKGATSTAAKYLAQTQN-ASDLIGGVS-
E. coli K12 AmiB                SIHADAA-PNRSATGASVWVLSNRRANSEMASWLEQHEK-QSELLGGAGD
B. subtilis 168 CwlB             SIHANAN-DSSSPNGSETYYDTTYQA------------------------
B. anthracis 4229 pXO2-42       SIHANGFNGNAHGTETFYYKAPTQKSNP----------------------
M. smegmatis mc2155 Ami2        SIRCATQ-PSPAANGVASFYFGNSHGSVS---------------------
M. tuberculosis H37Rv Ami2      SIRCETQ-TSLAANGVASFHFGNSHGSVS---------------------
M. leprae TN Ami2               SIRCETQ-TSPAANGVASFHFGNSHGSVS---------------------
M. tuberculosis H37Rv Ami1      SIHADGG--PASGRGFHVNYSAPPLNAIQ---------------------
M. leprae TN Ami1               SIHGDGG--PASGRGFHVIYSAPPLNPVQ---------------------
M. smegmatis mc2155 Ami1        SIHADGG--PPTGRGFHVLYSSPPLNAAQ---------------------
                                *::
                                (H415)
```

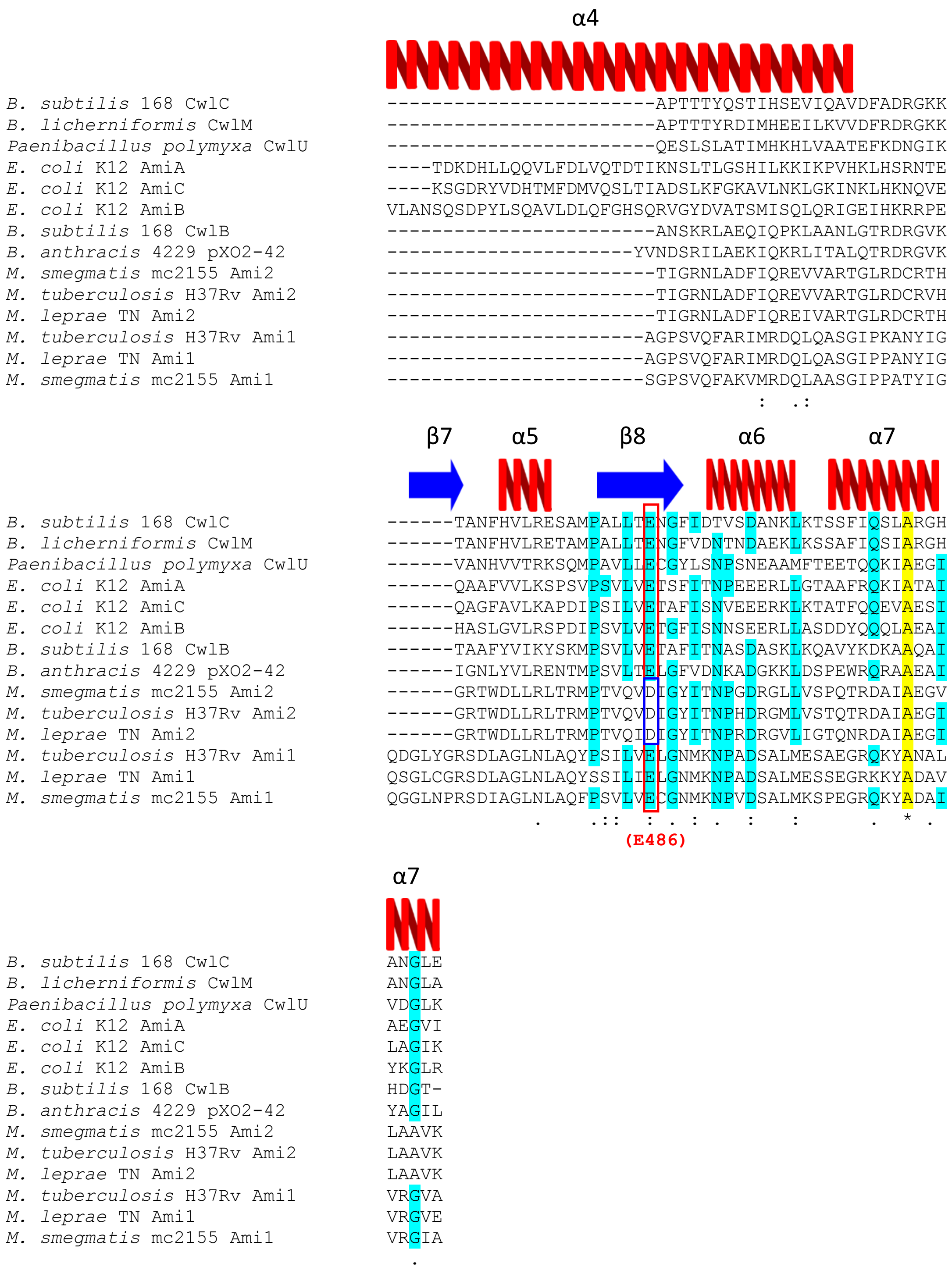
α4
B. subtilis 168 CwlC ------------------------APTTTYQSTIHSEVIQAVDFADRGKK
B. licherniformis CwlM ------------------------APTTTYRDIMHEEILKVVDFRDRGKK
Paenibacillus polymyxa CwlU ------------------------QESLSLATIMHKHLVAATEFKDNGIK
E. coli K12 AmiA ----TDKDHLLQQVLFDLVQTDTIKNSLTLGSHILKKIKPVHKLHSRNTE
E. coli K12 AmiC ----KSGDRYVDHTMFDMVQSLTIADSLKFGKAVLNKLGKINKLHKNQVE
E. coli K12 AmiB VLANSQSDPYLSQAVLDLQFGHSQRVGYDVATSMISQLQRIGEIHKRRPE
B. subtilis 168 CwlB ------------------------ANSKRLAEQIQPKLAANLGTRDRGVK
B. anthracis 4229 pXO2-42 ----------------------YVNDSRILAEKIQKRLITALQTRDRGVK
M. smegmatis mc2155 Ami2 ------------------------TIGRNLADFIQREVVARTGLRDCRTH
M. tuberculosis H37Rv Ami2 ------------------------TIGRNLADFIQREVVARTGLRDCRVH
M. leprae TN Ami2 ------------------------TIGRNLADFIQREIVARTGLRDCRTH
M. tuberculosis H37Rv Ami1 -----------------------AGPSVQFARIMRDQLQASGIPKANYIG
M. leprae TN Ami1 -----------------------AGPSVQFARIMRDQLQASGIPPANYIG
M. smegmatis mc2155 Ami1 -----------------------SGPSVQFAKVMRDQLAASGIPPATYIG
: .:
β7 α5 β8 α6 α7
B. subtilis 168 CwlC ------TANFHVLRESAMPALLTENGFIDTVSDANKLKTSSFIQSLARGH
B. licherniformis CwlM ------TANFHVLRETAMPALLTENGFVDNTNDAEKLKSSAFIQSIARGH
Paenibacillus polymyxa CwlU ------VANHVVTRKSQMPAVLLECGYLSNPSNEAAMFTEETQQKIAEGI
E. coli K12 AmiA ------QAAFVVLKSPSVPSVLVETSFITNPEEERLLGTAAFRQKIATAI
E. coli K12 AmiC ------QAGFAVLKAPDIPSILVETAFISNVEEERKLKTATFQQEVAESI
E. coli K12 AmiB ------HASLGVLRSPDIPSVLVETGFISNNSEERLLASDDYQQQLAEAI
B. subtilis 168 CwlB ------TAAFYVIKYSKMPSVLVETAFITNASDASKLKQAVYKDKAAQAI
B. anthracis 4229 pXO2-42 ------IGNLYVLRENTMPSVLTELGFVDNKADGKKLDSPEWRQRAAEAI
M. smegmatis mc2155 Ami2 ------GRTWDLLRLTRMPTVQVDIGYITNPGDRGLLVSPQTRDAIAEGV
M. tuberculosis H37Rv Ami2 ------GRTWDLLRLTRMPTVQVDIGYITNPHDRGMLVSTQTRDAIAEGI
M. leprae TN Ami2 ------GRTWDLLRLTRMPTVQIDIGYITNPRDRGVLIGTQNRDAIAEGI
M. tuberculosis H37Rv Ami1 QDGLYGRSDLAGLNLAQYPSILVELGNMKNPADSALMESAEGRQKYANAL
M. leprae TN Ami1 QSGLCGRSDLAGLNLAQYSSILIELGNMKNPADSALMESSEGRKKYADAV
M. smegmatis mc2155 Ami1 QGGLNPRSDIAGLNLAQFPSVLVECGNMKNPVDSALMKSPEGRQKYADAI
. .:: : . : . : : . * .
(E486)
α7
B. subtilis 168 CwlC ANGLE
B. licherniformis CwlM ANGLA
Paenibacillus polymyxa CwlU VDGLK
E. coli K12 AmiA AEGVI
E. coli K12 AmiC LAGIK
E. coli K12 AmiB YKGLR
B. subtilis 168 CwlB HDGT-
B. anthracis 4229 pXO2-42 YAGIL
M. smegmatis mc2155 Ami2 LAAVK
M. tuberculosis H37Rv Ami2 LAAVK
M. leprae TN Ami2 LAAVK
M. tuberculosis H37Rv Ami1 VRGVA
M. leprae TN Ami1 VRGVE
M. smegmatis mc2155 Ami1 VRGIA
.

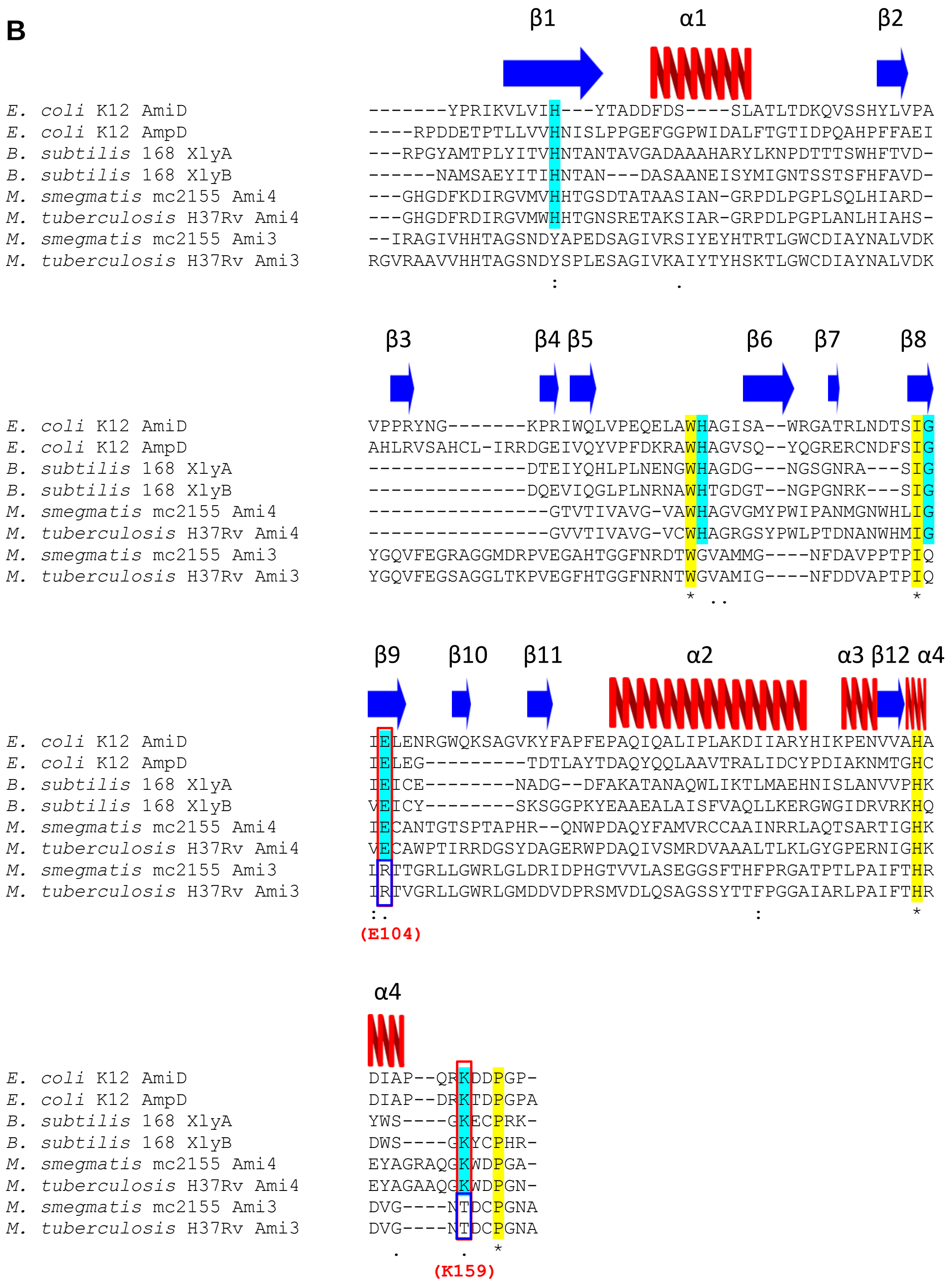

Figure S1: Amino acid alignments of amidases from various organisms. Alignments were carried out using ClustalW (http://www.ebi.ac.uk/Tools/msa/clustalw2/). Conserved domains are highlighted in yellow (100% homologous) and turquoise (≥ 70% homologous). **A.** Alignment of amidase_3 domains, red boxes and red bold text indicate conserved catalytic H341, E355, H415 and E486 residues as well as the mycobacterial H341R, H415R and E486D substitutions in Ami2 (blue boxes). **B.** Alignment of amidase_2 domains conserved catalytic residues (red boxes, red bold text). Shown are the conserved E104 and K159 catalytic residues in *ami4* as well as the E104P and K159T substitutions in *ami3* (blue boxes).

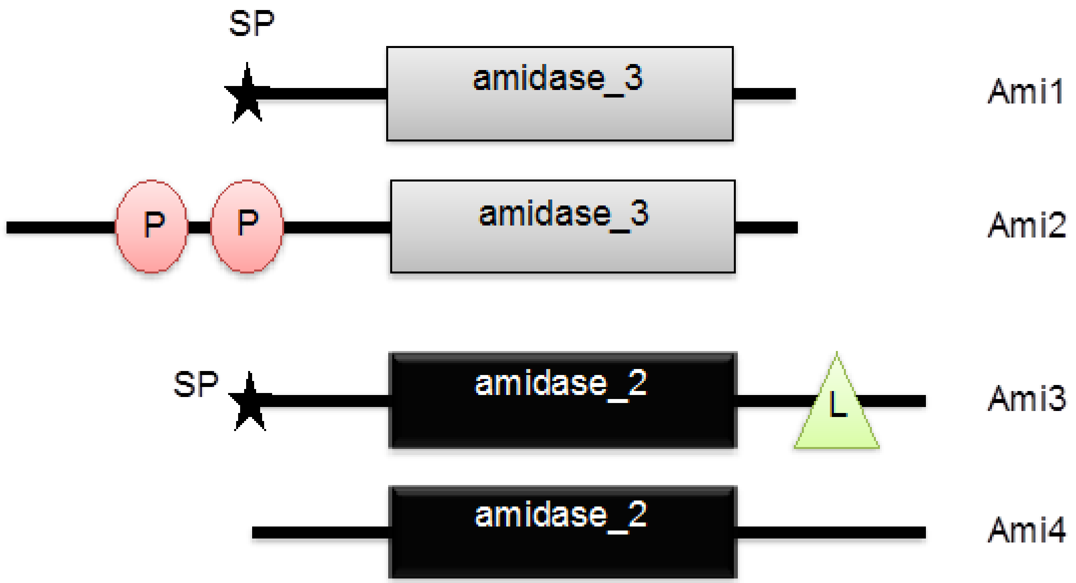

Figure S2: Diagrammatic representation of the four mycobacterial amidases. Shown are the amidase domains and any additional features identified through bioinformatics using the Biocyc (http://www.biocyc.com/ecocyc/index.shtml) and SignalP (http://www.cbs.dtu.dk/services/SignalP/) databases. P: peptidoglycan binding domain, SP: signal peptide and L: transmembrane domain.

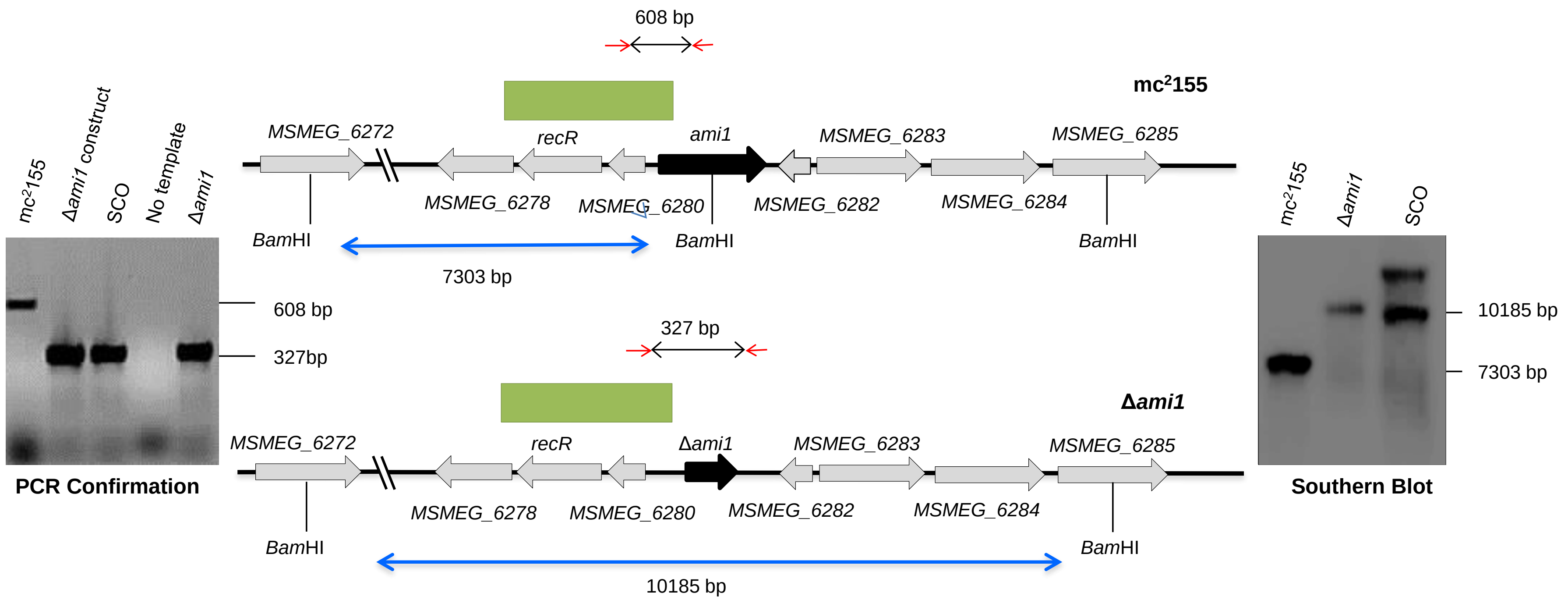

Figure S3. Genotypic analysis of the Δ*ami1* deletion strain by PCR and Southern blot analysis. The genomic map of the relevant locus is shown for the wild type mc^{2}155 and Δ*ami1* strains. Also shown on the left is the PCR confirmation of the genotype and Southern Blotting is shown on the right. For PCR confirmation, chromosomal DNA was used to amplify the *ami1* alleles from the wild type and mutant strains using the primer pairs described in Table S3 and indicated by red arrows above. The expected sizes of the amplicons are as follows: *ami1* - 608 bp and Δ*ami1* - 327 bp. For Southern blot analysis, chromosomal DNA from the Single crossover (SCO), parental and mutant strain was digested with *Bam*HI. The probe used for hybridization is shown as a solid green box and the expected sizes are indicated by the blue arrows. Maps are not drawn to scale.

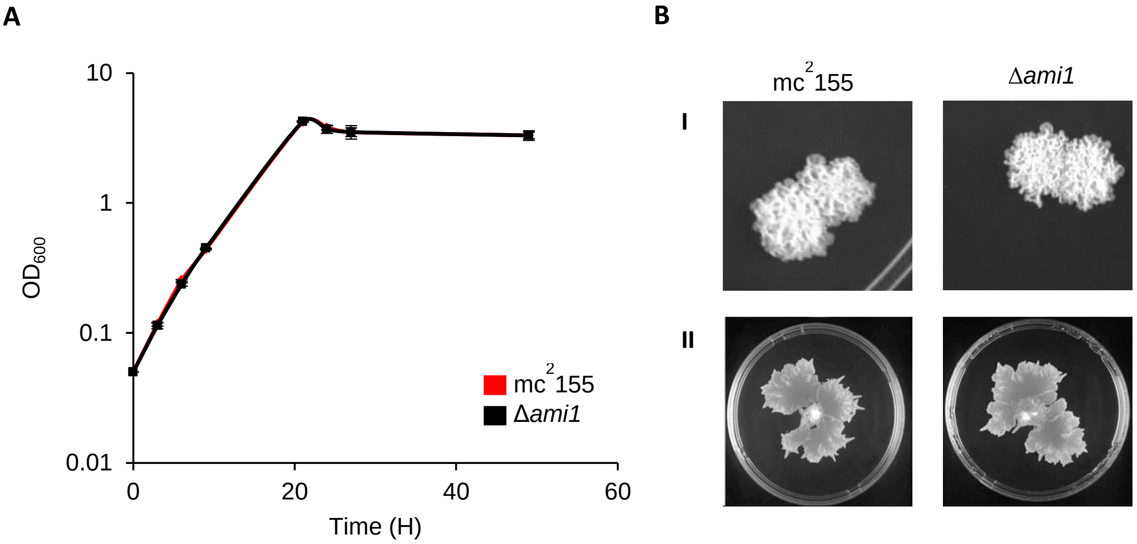

Figure S4: Deletion of *ami1* does not affect growth kinetics, colony morphology and sliding motility. A. Growth curve depicting the growth kinetics of the mc^{2}155 and Δ*ami1* strains. Error bars represent the standard deviation of three experiments. B. (I) Colony morphology of mc^{2}155 and Δ*ami1* on 7H10 agar. (II) Sliding motility assays with the mc^{2}155 and Δ*ami1* strains. Shown is the sliding motility on M63 media containing 0.3 % agar.

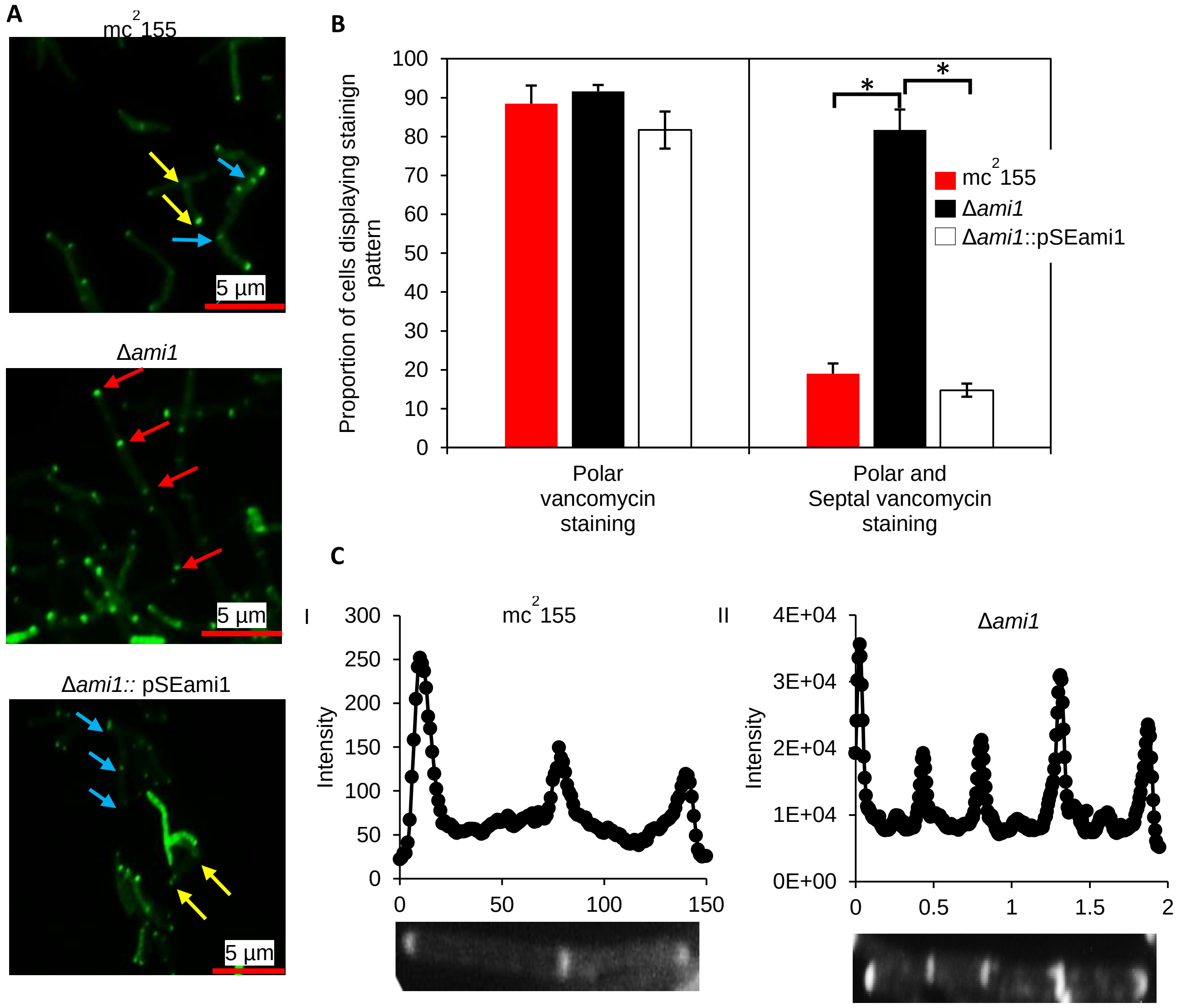

Figure S5: Fluorescence microscopy depicting the cellular incorporation of BODIPY-conjugated vancomycin in *M. smegmatis*. A. Subcellular localization of BODIPY-vancomycin in the mc^{2}155, Δ*ami1* and Δ*ami1*::pSEami1 strains. Also shown are the observed incorporation patterns, which included bipolar (yellow arrows); bipolar and septal (blue arrows) and bipolar with multiple septa (red arrows). B. Histogram depicting the proportion of cells displaying polar or polar and septal incorporation patterns. *: $p < 0.0001$. C: Distribution of the fluorescence intensity across the lateral axis of the *M. smegmatis* cell. I: mc^{2}155 and II: Δ*ami1*.

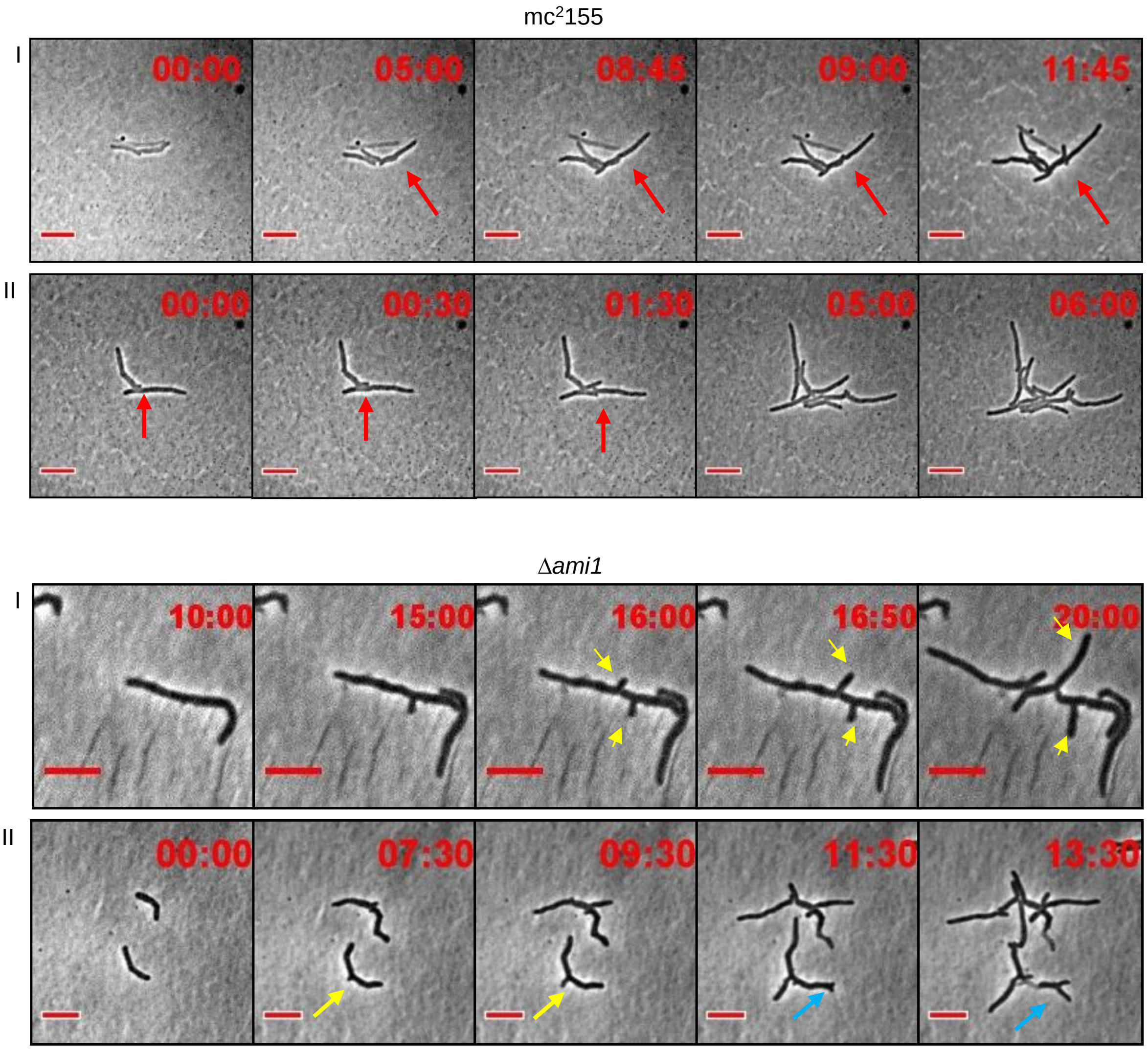

Figure S6: Time-lapse microscopic analysis of mc^{2}155 and Ami1-defective cells. Cells were grown and imaged in 7H9 media. Red arrows depict polar extension of cells. Yellow arrows depict lateral budding and Y-formed cell division is shown by the blue arrows. I-II depicts two representative micro-colonies for each strain. Scale = 5 µm. Time frame is given in hours.

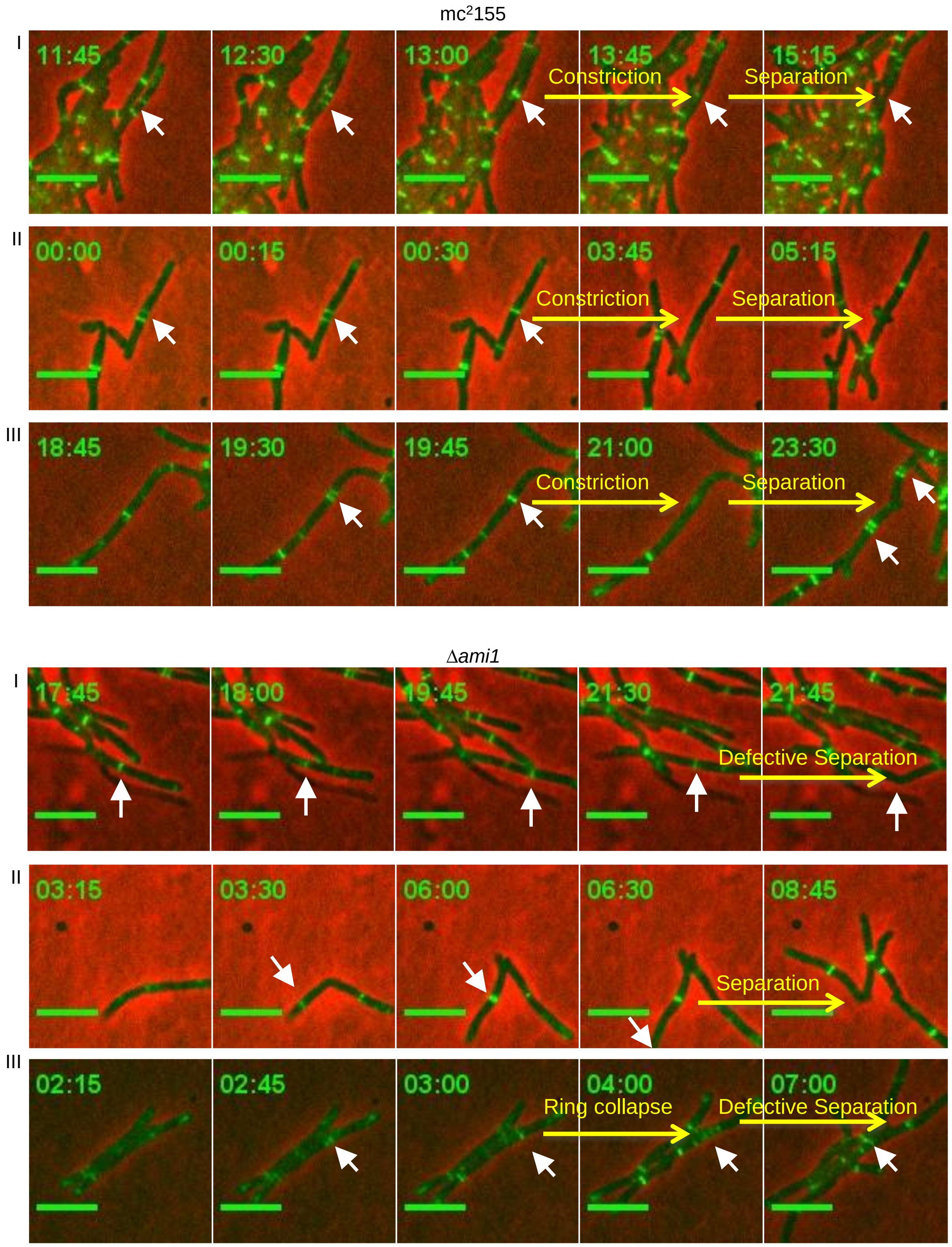

Figure S7. Time-lapse microscopic analysis showing the localization of FtsZ-rsEGFP in *M. smegmatis*. Cells were grown in Middlebrook 7H9 and subjected to real-time analysis of FtsZ bundling and/or constriction, indicated by white arrows. A, I-IV depict representative micro-colonies for the wild type and B, I-IV depict representative micro-colonies for the Δ*ami1* mutant. Scale bar = 5 µm. Time frame is given in hours.

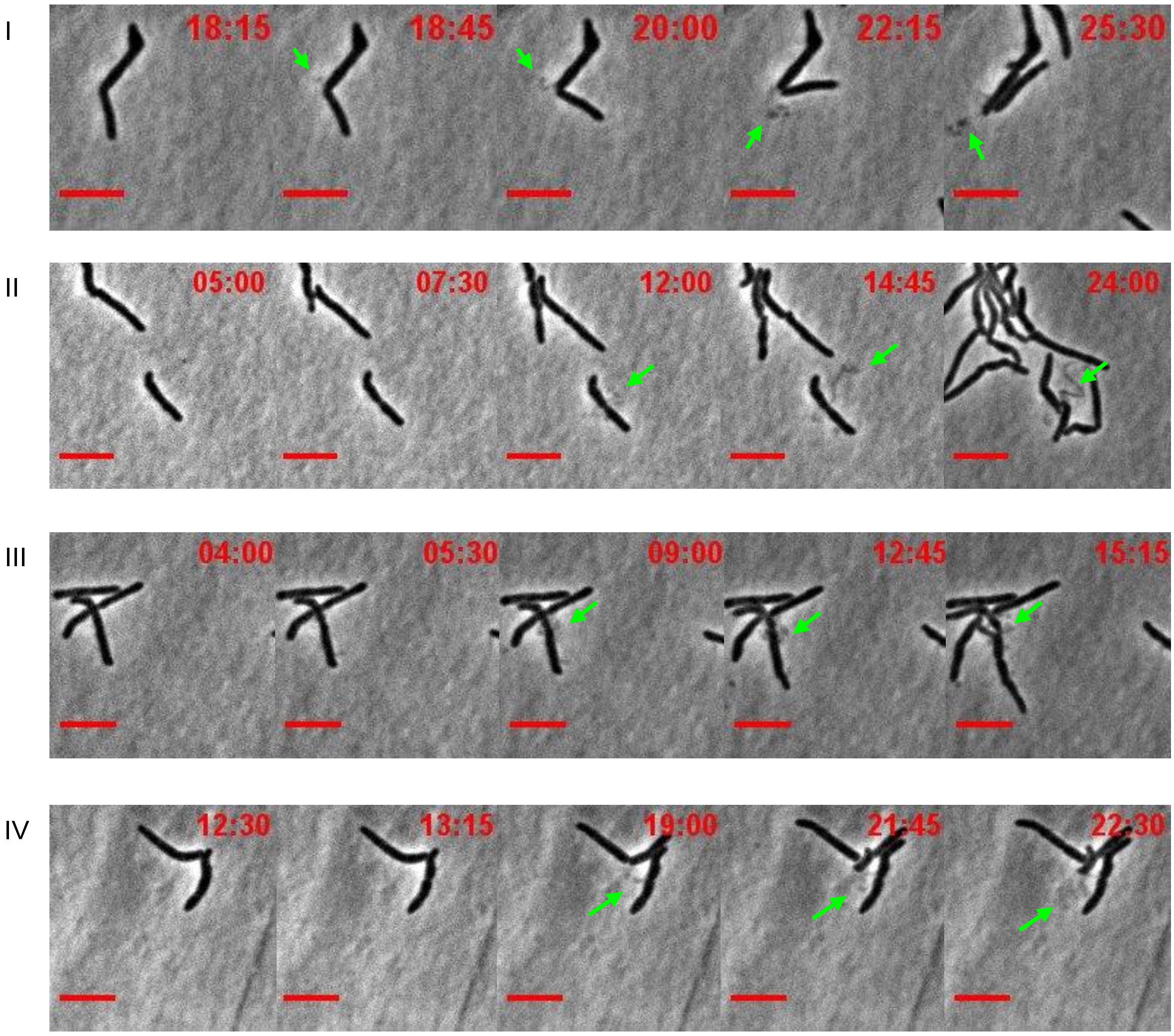

Figure S8: Time-lapse microscopic analysis of the Δ*ami1* deletion strain. Cells were grown and imaged in 7H9 media. I-IV depicts four micro-colonies that displayed septal cell envelope shedding as indicated by the green arrows. Scale = 5 µm. Time frame is given in hours.

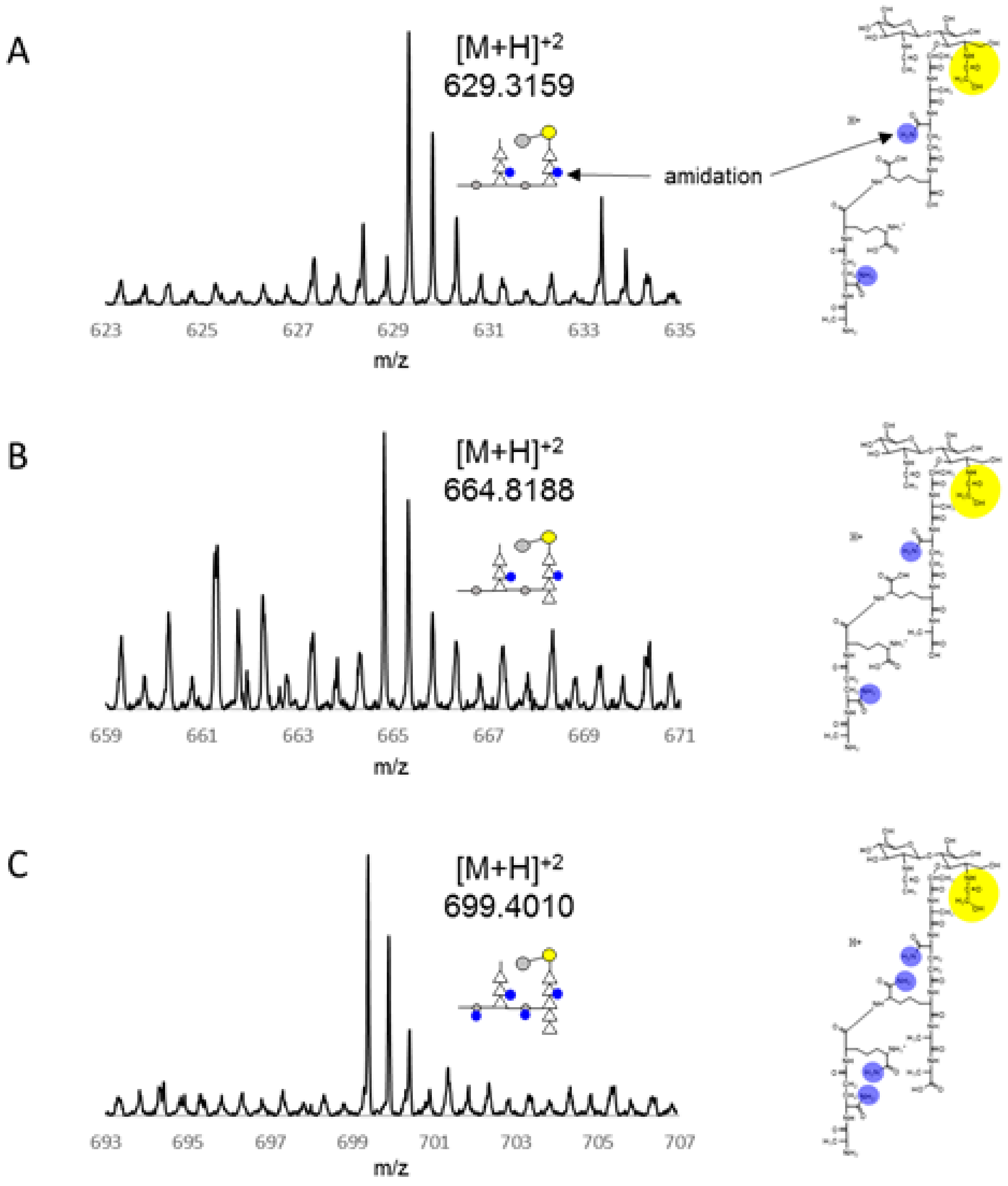

Figure S9. MS spectra of 3-3 cross-linked PG dimers from mc[2]155 with varying chemical modifications. A. Tripeptide-PG dimer with one disaccharide cleaved, a MurNGlyc, and two amidations. B. Tetrapeptide-PG dimer with one disaccharide cleaved, a MurNGlyc, and two amidations. C. Pentapeptide-PG dimer with one disaccharide cleaved, a MurNGlyc, and four amidations. Glyc – indicates glycolylation of the MurNAc

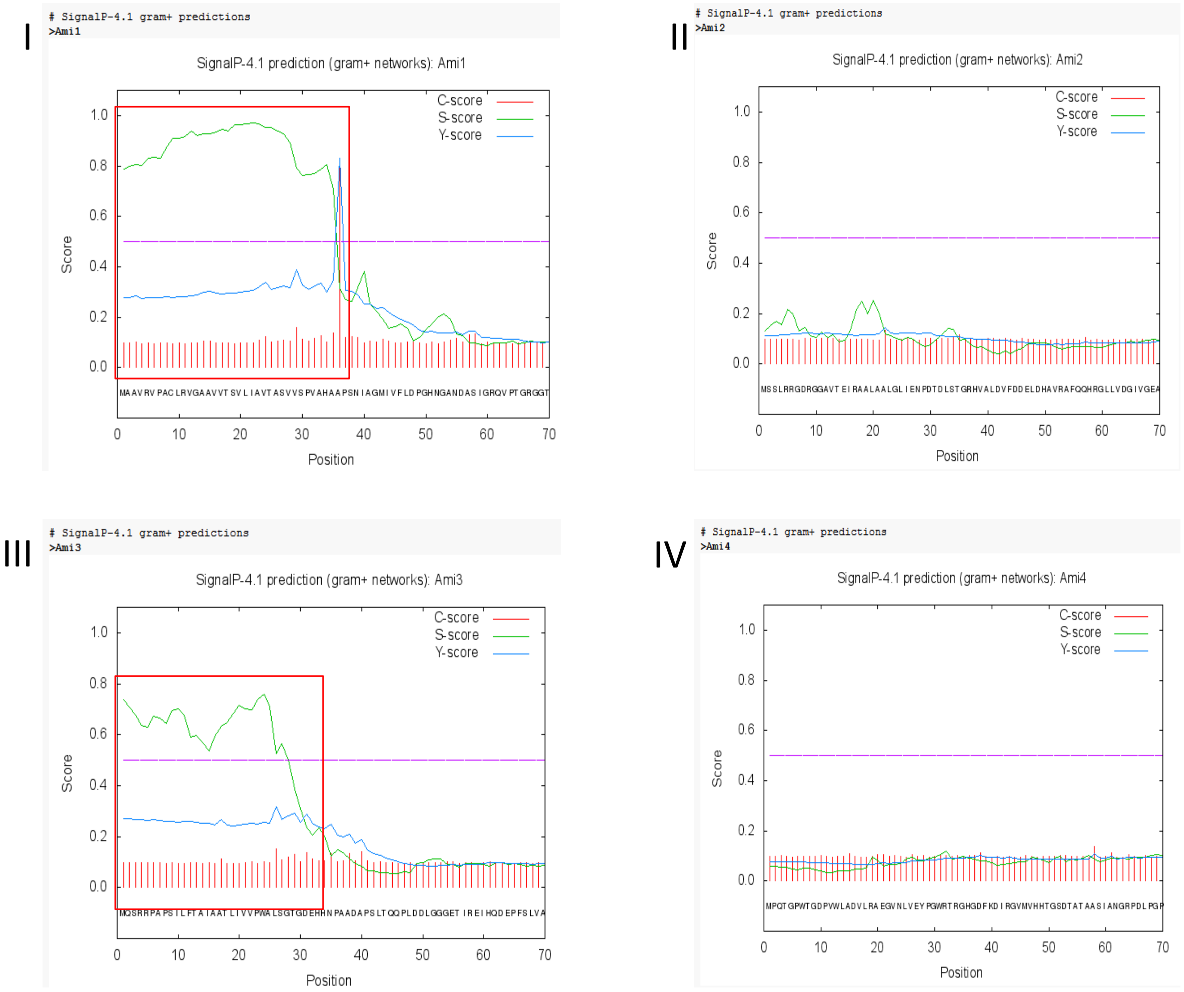

Figure S10. Signal sequence predictions in amidases from *M. smegmatis*. Red boxes indicate the presence of a signal sequence. Panels I-IV depict signal sequence analysis of Ami1-Ami4 respectively. Polypeptide amidase sequences were submitted to the Signal-P online tool assessing for the presence of both gram positive and gram negative specific signal sequences (http://www.cbs.dtu.dk/services/SignalP/). The SignalP tool produces three scores for each peptide bond, termed the C, S and Y scores. The C- and S-scores represent the output from the CS and SP network algorithms used to determine the cleavage site and to differentiate between signal peptide and the mature protein respectively. The Y-score is a combination of both C- and S-scores. An S-score value of 0.5 and above is used as a positive predictor for the presence of a signal sequence

Supplementary Movie Legends

Supplementary Movie S1. Single-cell time-lapse microscopy of wild type *M. smegmatis* mc^{2}155. Cells were grown in Middlebrook 7H9 medium and exhibited typical mycobacterial growth, involving cell elongation and cell separation resulting in two daughter cells (scale bar = 5µm).

Supplementary Movie S2. Single-cell time-lapse microscopy of the *M. smegmatis* Δ*ami1* mutant. Cells were grown in Middlebrook 7H9 medium and exhibited abnormal mycobacterial growth, involving the formation of ectopic lateral branches along the longitudinal axis of the cell. Also shown is the formation of buds that allow for Y-form cell division (scale bar = 5µm).

Supplementary Movie S3. Single-cell time-lapse microscopy of DivIVA localization in wild type *M. smegmatis* mc^{2}155. Cells were grown in Middlebrook 7H9 medium. Shown is the polar localization of DivIVA during cell elongation followed by septal localization prior to septum cleavage (scale bar = 5µm).

Supplementary Movie S4. Single-cell time-lapse microscopy of DivIVA localization, in the septal region, of the *M. smegmatis* Δ*ami1* mutant. Cells were grown in Middlebrook 7H9 medium. Depicted is the polar localization of DivIVA during cell elongation followed by septal localization, however, lack of septum cleavage resulted in flickering of the DivIVA foci, leading to the formation of an ectopic growth pole at septum, which subsequently matured into a lateral branch (scale bar = 2µm).

Supplementary Movie S5. Single-cell time-lapse microscopy of DivIVA, along the lateral axis of the cell, in the *M. smegmatis* Δ*ami1* mutant. Cells were grown in Middlebrook 7H9 medium. The aberrant placement of DivIVA foci during cell elongation, along the lateral axis of the cell, is shown. This was followed by the formation of an ectopic growth pole, which subsequently matured into a lateral branch (scale bar = 2µm).

Supplementary Movie S6. Single-cell time-lapse microscopy of FtsZ localization in wild type *M. smegmatis* mc^{2}155. Cells were grown in Middlebrook 7H9 medium. The localization of two FtsZ rings at sites adjacent to the future septum, followed by ring coalescence at the septum is depicted. This is followed by ring constriction and subsequent cell separation (scale bar = 5µm).

Supplementary Movie S7. Single-cell time-lapse microscopy of FtsZ localization in the *M. smegmatis* Δ*ami1* mutant. Cells were grown in Middlebrook 7H9 medium. Shown is the localization of an FtsZ ring at the septum resulting in 3 possible outcomes: (I) The complete collapse of the FtsZ ring, which occurs upon failure to degrade the septum, (II) The collapse and reformation of the ring at the same site, also occurring due to failed septum degradation or (III) Constriction of the ring, resulting in cell separation (scale bar = 5µm).

Supplementary Movie S8. Single-cell time-lapse microscopy of the *M. smegmatis* Δ*ami1* mutant depicting release of material from the septum. Cells were grown in Middlebrook 7H9 medium. Shown is the release of material from the septum (scale bar = 2µm).

Supplementary Movie S9. Single-cell time-lapse microscopy of septal cell wall release in the *M. smegmatis* Δ*ami1* mutant. Cells were grown in Middlebrook 7H9 medium and stained with membrane stain FM4-64 (scale bar = 2µm).

References

1 Snapper, S. B., Melton, R. E., Mustafa, S., Keiser, T. & Jacobs Jr, W. R., Jr. . Isolation and Characterization of efficient plasmid transformation mutants of *Mycobactrium smegmatis*. *Mol Microbiol* **4**, 1911-1919 (1990).

2 Parish, T. & Stoker, N. G. Use of a flexible cassette method to generate a double unmarked Mycobacterium tuberculosis tlyA plcABC mutant by gene replacement. *Microbiology* **146 (Pt 8)**, 1969-1975 (2000).

3 Ehrt, S. *et al.* Controllong gene expression in mycobacteria with anhydrotetracycline and Tet repressor. *Nucleic Acids Res* **33**, e21 (2005).

4 Santi, I., Dhar, N., Bousbaine, D., Wakamoto, Y. & McKinney, J. D. Single-cell dynamics of the chromosome replication and cell division cycles in mycobacteria. *Nature communications* **4**, 2470, doi:10.1038/ncomms3470 (2013).
